# Personalized Mobile App–Based Messaging and Step Tracking for Improving Walking Behavior in Adults: Randomized Controlled Trial

**DOI:** 10.2196/84430

**Published:** 2026-09-17

**Authors:** Sarang Jang, Aeree Sohn

**Affiliations:** 1 Department of Public Health Sahmyook University Seoul Republic of Korea

**Keywords:** digital health, mobile applications, mobile apps, personalized messaging, theory-based intervention, walking

## Abstract

**Background:**

Walking is one of the most effective forms of physical activity; however, maintaining long-term engagement in digital step-counting interventions remains challenging.

**Objective:**

This study aimed to evaluate the effects of personalized message interventions integrating the Information-Motivation-Behavioral Skills (IMB) model and regulatory focus theory (RFT) on walking behavior.

**Methods:**

Participants were recruited offline through community advertisements containing a QR code that linked to an online sign-up form and were screened using an online baseline questionnaire by research staff, while all intervention delivery and outcome assessments were conducted via the WalkOn mobile app. A 12-week randomized controlled trial targeting 174 adults in Mapo-gu, Seoul, Korea, was conducted from April 1 to June 22, 2024. Of the 174 randomized participants, 141 (67 in the intervention group and 74 in the control group) engaged in the intervention. Both groups received incentives based on goal achievement and general health information, whereas the intervention group received weekly personalized messages provided by research staff according to midweek step-count performance. Primary outcomes included weekly goal achievement rates for the 25,000-step goal, step-count patterns, and consecutive 2-week goal achievement failure events. Analyses were conducted using generalized estimating equations (GEEs), linear mixed models (LMMs), and Cox proportional hazards models.

**Results:**

Of the 174 participants randomized, 141 (81%) engaged in the intervention and were included in the analyses (67 intervention; 74 control), while 33 participants did not participate after randomization. The overall 12-week mean goal achievement rate was 87% in the intervention group and 87% in the control group, with no significant difference between the groups (odds ratio [OR] 1.12, 95% CI 0.29-4.37; *P*=.87). Weekly step-count patterns also showed no significant differences (group × time interaction: estimate=–417; SE 09; *P*=.31). However, the risk of consecutive 2-week goal achievement failure events was significantly lower in the intervention group (hazard ratio [HR] 0.67, 95% CI 0.52-0.86; *P*=.002). The intervention group showed an approximately 33% lower likelihood of consecutive 2-week goal achievement failure events than the control group.

**Conclusions:**

Personalized message interventions did not show significant effects on overall step-count increase but were associated with a reduced risk of consecutive 2-week goal achievement failure events. These findings suggest that personalized messages may play an important role in improving behavior change sustainability and failure resilience. Incorporating personalized messaging strategies may enhance the effectiveness of digital health intervention design.

**Trial Registration:**

Clinical Research Information Service (CRIS), Republic of Korea KCT0010966; https://tinyurl.com/3jeepxve

## Introduction

Walking is one of the most accessible and effective forms of physical activity and is consistently associated with reduced risks of cardiovascular disease, cancer, type 2 diabetes, and dementia [1-3]. In particular, according to meta-analyses and large-scale cohort studies, walking briskly for 30 minutes daily, 5 days a week, has been shown to significantly reduce cardiovascular disease risk factors, improve blood pressure control, and contribute to healthy aging [1,2,4]. Additionally, walking for 30 minutes daily is associated with substantial health benefits, including improved mental health, enhanced mobility, and increased life expectancy [5]. Walking interventions have been emphasized in rehabilitation contexts and programs for older adults, and structured and sustained walking programs have shown particularly important benefits for mobility, balance, quality of life, and stroke recovery [6]. Recent studies have shown that even short walks of 5 to 11 minutes per day are associated with reduced mortality from cancer, cardiovascular disease, and all causes [7]. Walking is an ideal tool for population-level health promotion interventions due to its accessibility and scalability, as it requires no special equipment, can be performed in various environments, and can be adapted to an individual’s fitness level [8].

Despite these clear health benefits, physical inactivity remains a serious public health problem worldwide, contributing to the burden of noncommunicable diseases and reduced quality of life [9]. As an innovative approach to address this issue, digital step-counting interventions using pedometers, smartphone apps, and wearable devices have emerged as promising strategies to encourage walking behavior and increase overall physical activity levels. These interventions have received significant attention owing to their accessibility, cost-effectiveness, and scalability across diverse populations.

The proliferation of digital health technologies has created unprecedented opportunities for individuals to monitor their physical activity on their own [10], and step counters provide real-time feedback on daily activity levels and enable users to track their progress toward the recommended physical activity guidelines. The appeal of such interventions lies in the simplicity and quantifiability of step counting, which can serve as a visible and motivating goal for individuals seeking to increase their physical activity.

There is growing evidence supporting the short-term effectiveness of digital step counting in promoting actual walking behavior. According to several systematic reviews and meta-analyses, such interventions significantly increased daily step counts, with an average increase of 1126 steps per day after 4 months of pedometer use, and sustained improvements were observed even up to 3 to 4 years later [11]. Similarly, interventions using smartphone apps have demonstrated an average daily increase of 1182 steps over 3 months [12]. These increases are often accompanied by meaningful improvements in health outcomes such as weight loss, reduced BMI, lower blood pressure, decreased triglyceride levels, and improved psychological well-being [12,13].

However, despite these initial positive results, there is growing evidence that digital step-counting interventions have significant limitations in maintaining long-term walking behavior changes. The novelty and enthusiasm surrounding these technologies tend to wane over time, leading to lower participation and adherence rates. Several longitudinal studies have reported that more than half of the participants discontinue device use within 6 months of a digital step-counting intervention [14]. The novelty effect begins to fade after approximately 3 months, with the effect size in long-term interventions decreasing by approximately 40% compared with short-term interventions [15]. These sustainability issues highlight the significant gap between the short- and long-term public health impacts, suggesting that maintaining high activity levels requires addressing complex psychological, social, and environmental factors beyond simple self-monitoring.

To overcome these limitations, digital walking interventions have evolved around step-counting technology using wearable devices and mobile apps, with various efforts to increase physical activity engagement [16]. Current digital walking interventions commonly integrate behavior change techniques (BCTs) such as self-monitoring, goal setting, social comparison, and gamification, which are increasingly enhanced through personalized feedback [17,18]. Among these approaches, personalized messaging has proven to be a particularly effective form of personalized feedback that supports behavior maintenance by providing tailored information and motivation based on individual behavioral data [19]. Research shows that personalized messages elicit higher levels of engagement and adherence than generic information approaches [20,21].

However, current digital walking interventions have several limitations that restrict their effectiveness. First, the accuracy of commercially available step counters can vary significantly in real-world settings, with limited representativeness and generalizability across diverse populations [22,23]. Second, user engagement and compliance tend to decrease over time, and the long-term maintenance of behavior change remains an ongoing challenge [11,24]. Third, existing personalized messaging research primarily focuses on general encouragement or simple performance feedback and lacks systematic research on differentiated theory-based messaging strategies according to individual real-time performance levels. Fourth, most studies rely on single behavior change theories, failing to comprehensively address individuals' complex motivations and change stages. These limitations emphasize the need for more sophisticated intervention approaches beyond simple feedback mechanisms.

Digital health interventions are increasingly integrating behavior change theories to improve their effectiveness [25,26]. While the effectiveness of an intervention depends on the appropriateness of the theoretical framework rather than the mere number of theories used, this study specifically integrates the Information-Motivation-Behavioral Skills (IMB) model and regulatory focus theory (RFT) to support the design of personalized walking interventions. Existing research is limited to adaptive approaches that comprehensively address information provision, motivation, and behavioral skill acquisition while dynamically adjusting prevention- or promotion-focused messages according to individual weekly performance changes.

The IMB model proposes that three core components are necessary for behavior change [27]: (1) accurate information related to the behavior, (2) personal and social motivation to perform the behavior, and (3) behavioral skills to effectively perform the behavior [28,29]. This model provides a structured theoretical framework supporting users in understanding relevant information, developing intrinsic and extrinsic motivation, and acquiring the skills necessary for behavior change. Similarly, the RFT explains that individual motivational orientations are distinguished as prevention and promotion foci [30-32]. RFT proposes that individuals regulate goal pursuit through 2 distinct motivational orientations: promotion focus and prevention focus [33,34]. A promotion focus is concerned with aspirations, accomplishments, and advancement, motivating individuals to pursue gains and ideal outcomes. In contrast, a prevention focus centers on safety, responsibility, and the fulfillment of obligations, where the primary motivation is loss avoidance and the maintenance of a non-loss state. This theory can support habit formation by effectively encouraging the repetition and habituation of walking by connecting goal achievement with positive reinforcement and rewards.

However, the IMB model has limitations in that it does not consider differences in individual motivational orientations and regulatory focus types and fails to explain dynamic changes in motivational needs according to performance levels [35,36]. Conversely, RFT has limitations in that it does not provide specific behavior change mechanisms, focuses primarily on motivational orientation, and does not specify what types of information or skills should be provided according to the regulatory focus [33,37]. These limitations of each theory can be addressed through complementary integration.

The integration of the IMB model and RFT operates through integrative processes that mutually complement the limitations of each theory. This study proposes an integrative approach that differentiates the IMB’s information component into loss or gain frames according to RFT’s prevention and promotion foci, applies motivational strategies tailored to individual regulatory focus, and provides behavioral skills differentiated by regulatory focus (prevention focus: barrier removal; promotion focus: performance optimization). Conversely, the IMB model provides a foundation for integrating systematic information provision mechanisms, specific behavioral skill acquisition processes, and multilayered motivational systems to complement RFT’s limitations.

Specifically, this study proposes a performance-based dynamic integration model that dynamically determines the regulatory focus based on participants’ real-time step count performance and selectively applies IMB components accordingly. Low-performance groups (<12,500 steps) activate a prevention focus emphasizing information and behavioral skills, medium-performance groups (12,500-24,999 steps) apply a prevention focus and an IMB integrated approach, and high-performance groups (≥25,000 steps) activate a promotion focus emphasizing motivation.

Based on these theoretical and practical needs, this study aims to overcome existing research limitations and present new approaches. The originality of this study lies in the following aspects. First, we developed a personalized messaging system that is responsively adjusted weekly based on the participants’ real-time step count performance. Second, we systematically implemented differentiated messaging strategies according to individual performance levels by applying a theoretical framework that integrates the IMB model and RFT. Third, we comprehensively evaluated the intervention effects from the perspective of behavior change maintenance, focusing on goal achievement sustainability and consecutive failure prevention beyond a simple step count increase. This approach overcomes the limitations of existing static and uniform personalized messaging and presents the possibility of dynamic, customized interventions tailored to individual change stages and performance levels.

Therefore, this study seeks to answer the following specific research questions: (1) Do personalized theory-based messages have significant effects on weekly step count goal (25,000 steps) achievement rates over 12 weeks? (2) Does the message intervention have significant effects on participants’ step count patterns over 12 weeks? and (3) Do personalized messages reduce the risk of consecutive 2-week goal achievement failures? To answer these research questions, this study adopted a randomized controlled trial (RCT) design and conducted a 12-week digital walking intervention targeting 174 adults.

## Methods

### Study Design

This study was a 12-week RCT conducted from April 1 to June 22, 2024, to evaluate the effects of personalized health message interventions on walking behavior in Korean adults. A parallel-group design with a 1:1 allocation ratio was used. An active control group design was adopted to isolate the specific effects of personalized messaging by providing substantial nonpersonalized intervention components to the control group.

The trial was registered retrospectively with the Clinical Research Information Service (CRIS; KCT0010966). The registered study procedures corresponded to the protocol implemented in the trial. The study protocol, including primary outcomes and statistical analysis plans, was finalized prior to data analysis. No changes were made to the primary outcomes or analytical strategies after examination of the data. The registered protocol corresponds to the procedures implemented in the trial.

### Sample Size

The sample size calculation was performed using G*Power. Assuming a small effect size (Cohen *d*=0.20), a significance level of α=0.05, and power of 80%, the required sample size for the primary comparison was 200 participants per group. However, the final number of participants randomized was 174, with 141 engaging in the study (67 in the intervention group and 74 in the control group). The impact of this reduced sample size on statistical power and precision is discussed in the Discussion section.

### Study Participants

#### Overview

Residents of Mapo-gu, Seoul, were recruited through voluntary participation via community offline advertisements (eg, local posters and bulletin boards containing a QR code that linked to an online sign-up form) and were screened using an online baseline questionnaire by research staff. Eligibility criteria included being an adult aged 19 years or older, owning a smartphone capable of installing a mobile app, being able to walk independently, and having signed a consent form for study participation. Those with difficulty walking or an inability to use a smartphone app were excluded. A total of 174 participants were randomized (87 per group).

Participants received incentive points for achieving weekly walking goals during the intervention period. The incentive value progressively increased every 4 weeks (weeks 1-4: 1000 points [KRW 1000; KRW 1=US $0.00074 as of April 5, 2024]; weeks 5-8: 1500 points [KRW 1500]; weeks 9-12: 2000 points [KRW 2000]) to encourage sustained participation.

#### Randomization and Intervention

After baseline data collection, the participants were assigned to either the intervention or control group using a computer-generated randomization sequence created with the RAND function in Microsoft Excel. Following randomization, the intervention began, and participants were monitored throughout the 12-week intervention period. Both groups were presented with a weekly walking goal of 25,000 steps and used the WalkOn mobile app (Swallaby Co) for real-time step tracking.

The intervention was implemented using the WalkOn mobile app, a commercially available smartphone app that tracks users’ daily step counts using built-in motion sensors and provides real-time feedback on walking activity. Step count data were automatically recorded and stored on the system server, allowing researchers to collect participants’ walking data throughout the 12-week intervention period. Program participation was defined as having at least one recorded step count through the WalkOn mobile app after the intervention began. Participants who had no recorded step count data during the first week of the intervention were classified as nonparticipants and were considered not to have initiated the program. These participants had agreed to participate but had no recorded step-count data during the first week after randomization, indicating that the WalkOn step-tracking app had not been activated on their smartphones.

The weekly goal of 25,000 steps established in this study was set by converting the World Health Organization’s (WHO) physical activity recommendations for adults—“at least 150 minutes of moderate-intensity physical activity per week”—into step counts [34]. According to previous research, 30 minutes of moderate walking corresponds to approximately 3000 steps when performed at a walking cadence of approximately 100 steps per minute [38,39]. Multiple controlled laboratory studies have confirmed that a cadence of 100 steps per minute corresponds to moderate physical activity intensity (3 metabolic equivalents [METs]) [40,41]. In addition, step-count–based physical activity classifications suggest that fewer than 5000 steps per day represents a sedentary lifestyle threshold [42]. Accordingly, accumulating approximately 25,000 steps per week represents a realistic and attainable behavioral target that encourages participants to move beyond sedentary activity levels while remaining feasible for community-based populations.

Based on these considerations, this study set the weekly step goal at 25,000 steps, equivalent to approximately 5 days of moderate walking per week. This threshold also allowed meaningful midweek performance classification (<50%, 50%-99%, and ≥100%) for the personalized messaging algorithm used in the intervention. Therefore, the selected goal was considered both practically achievable and consistent with the WHO physical activity recommendation standards.

The goal was intentionally based on approximately 5 days of activity rather than 7 days to reflect the WHO recommendation structure (30 minutes of moderate physical activity on at least 5 days per week). In addition, allowing 2 potential rest days per week reflects realistic physical activity patterns in community populations and improves the feasibility and sustainability of behavioral interventions.

### Intervention Group (Personalized Message Group)

The intervention group received personalized health messages via KakaoTalk (Kakao Corp) every Friday based on walking performance from Monday to Wednesday of each week, in addition to basic intervention elements.

Participants’ performance groups were determined based on the cumulative step counts recorded from Monday to Wednesday of each week, rather than projected weekly averages. These midweek step totals were compared with the predefined weekly goal of 25,000 steps to classify participants into three performance categories (<50%, 50%-99%, and ≥100%). Personalized messages were developed using RFT and the IMB model.

Participants were classified into three subgroups based on the cumulative step counts recorded from Monday to Wednesday of each week: (1) fewer than 12,500 steps received prevention-focused messages emphasizing information and behavioral skills; (2) 12,500 to 24,999 steps received prevention-focused messages, including all 3 IMB model components; and (3) 25,000 steps or more (≥100% of the weekly goal) received promotion-focused messages emphasizing motivation. Participants who accumulated ≥25,000 steps between Monday and Wednesday were considered to have already achieved the weekly goal ahead of schedule and therefore received promotion-focused reinforcement messages designed to sustain their walking behavior for the remainder of the week. Motivational elements were present across the intervention through goal setting and incentive systems; however, message content for the low-performance group primarily emphasized information and behavioral skills to address practical barriers to walking. The performance categories were predefined prior to study implementation and were directly linked to the weekly goal of 25,000 steps and the associated incentive system. Because incentives were provided upon achieving the 25,000-step weekly target, proportional progress toward this predefined goal (<50%, 50%-99%, and ≥100%) was used to tailor intervention messages. Thus, the categorization reflected meaningful stages of goal attainment rather than arbitrary thresholds. The specific message strategies and theoretical components for each performance range are summarized in Table 1. Each message was personalized with the participants’ names and specific performance feedback, including concrete walking suggestions reflecting the seasonal context and local characteristics.

**Table 1:** Theory-based personalized message components and examples by performance group.

Performance group	Step count range	Theoretical framework	Message focus and example^a^
High-performance group	≥25,000 steps per week	RFT^b^: promotion focus IMB^c^: motivation	Focus: celebrating success and maintaining engagement Example: “Great job! You’ve already achieved 25,000+ steps. You’ve earned 1,500 points. Congratulations!”
Medium-performance group	12,500 to 24,999 steps per week	RFT: prevention focus IMB: information, motivation, and behavioral skills	Focus: emphasizing proximity to goal with specific behavioral suggestions Example: “Mid-week check: You’ve reached more than half of your weekly goal (25,000 steps). How about evening walks to enjoy cricket sounds?”
Low-performance group	<12,500 steps per week	RFT: prevention focus IMB: information and behavioral skills	Focus: providing specific behavioral strategies and preventing goal abandonment Example: “Did you know that just a 5-minute walk after meals can help lower your blood sugar and boost your mood? Try a short stroll today!”

^a^Messages were delivered every Friday via KakaoTalk to intervention group participants (n=67) based on Monday to Wednesday step count assessment. Each message included personalized greetings using participants’ names or nicknames.

^b^RFT: regulatory focus theory.

^c^IMB: Information-Motivation-Behavioral Skills.

### Control Group (Active Control Group)

The control group was designed as an active control group that received all intervention elements except personalized messages. Specifically, they received the following components:

- Same incentive system: both groups received the same progressive incentive system for achieving weekly walking goals during the intervention period.
- General health information provision:
  participants received standardized, nonpersonalized health information weekly in card news format, including information on walking, smoking cessation, alcohol moderation, and community health resources.
- Social comparison feature:
  participants could experience social comparison and competitive motivation through real-time step count comparison features within the WalkOn app.
- Same goal setting:
  participants received the same weekly walking goal of 25,000 steps as the intervention group.

Through this active control group design, we aimed to isolate the effect of personalized messages and evaluate the added value of personalization beyond general health information provision or social comparison. All intervention components except personalized messaging were identical between the 2 groups.

### Operational Definition of the Theoretical Framework

The IMB and RFT integrated framework developed in this study is a system that dynamically determines RFT’s prevention and promotion focus according to participants’ weekly step count performance and selectively combines and applies IMB model components (information, motivation, and behavioral skills) accordingly.

### Performance-Based Regulatory Focus Determination

The participants were classified into 3 groups based on their midweek step count, with the personalization algorithm dynamically determining the regulatory frame to maximize motivational relevance. For the low-performance group (<12,500 steps per week; <50% achievement), messages used a prevention focus to emphasize “loss avoidance,” highlighting potential negative health outcomes and the risk of failing the weekly commitment to prevent goal abandonment. The medium-performance group (12,500-24,999 steps per week; ≥50% achievement) received messages that maintained a prevention-oriented frame while emphasizing the possibility of achieving the weekly goal. Conversely, for the high-performance group (≥25,000 steps per week; 100% achievement), a promotion focus was applied to celebrate achievement and progress, focusing on positive gains and encouraging the pursuit of higher health aspirations. This dynamic application of RFT ensures that the motivational feedback remains tailored to the individual’s real-time behavioral state.

### IMB Component Application Strategy

The IMB model components were applied differently for each performance range. Low-performance ranges were centered on information and behavioral skills components, providing specific implementation methods and self-monitoring strategies. Medium performance ranges provided comprehensive support by integrating all information, motivation, and behavioral skills components. High-performance ranges were centered on motivation components to induce achievement recognition and continued participation.

The information components emphasized the importance of goal achievement and current status feedback for prevention focus, achievement results, and rewards for promotion focus. Motivation components used supportive keywords like “cheer up” and “let’s do this together” for prevention focus, and recognition keywords like “excellent” and “congratulations” for promotion focus. Behavioral skills components provided specific practice strategies for prevention focus, and performance maintenance and expansion strategies for promotion focus.

### Message Generation Algorithm

Personalized messages were automatically generated using the step-by-step algorithm.

### Message Generation Procedure

In the first step, participants’ weekly step count data (Monday to Wednesday step count sum, with Thursday 12 AM as the cutoff) were collected to classify performance ranges. Midweek (Monday to Wednesday) step count assessment was intentionally implemented as an early trajectory monitoring strategy to facilitate timely feedback within the same weekly goal cycle. Because the intervention was fundamentally structured around the predefined weekly target of 25,000 steps, midweek performance evaluation allowed differentiated messaging to support goal attainment before the week concluded, rather than serving as a replacement for the weekly outcome measure. The second step determined the regulatory focus (prevention or promotion) according to the performance ranges and selected the IMB components to apply. The third step generated message content according to the selected components and added personalization elements.

### Message Structure Template

All messages were structured identically: (1) greeting including the personal name, (2) specific step count feedback, (3) core message by performance range, (4) specific behavioral suggestions, and (5) encouragement and closing. Each message was limited to 150 to 200 characters to maintain conciseness while preserving a friendly and personal tone to strengthen relationships with the participants. Examples of the performance-level messages are provided in Multimedia Appendix 1.

### Application of Personalization Elements

All messages included the participants’ real names, actual step counts, and goal achievement rates. They also suggested seasonally appropriate activities (spring: cherry blossom walks; summer: early morning walks) and specific locations reflecting local characteristics (Hangang Park and World Cup Park). Differential messages were provided according to change patterns compared with the previous weeks: encouragement for improvement, new strategy suggestions for stagnation, and supportive messages for decline.

### Outcome Measurement

Baseline characteristics, including gender and age, were measured through preintervention surveys. Step count-related outcome variables were calculated using daily step count data automatically collected via the WalkOn app, which uses built-in smartphone accelerometers with weekly verification procedures to ensure data accuracy and integrity.

Although participants could withdraw from the WalkOn community at any time, the research team had securely exported and stored all step-count data for the 12-week intervention period in Microsoft Excel files before any withdrawals, ensuring that participant withdrawal did not affect the completeness or integrity of the final dataset.

### Weekly Goal Achievement Rate

Weekly goal achievement rate was defined as the proportion of participants achieving ≥25,000 steps per week over 12 weeks, with individual achievement coded as binary (1=achievement; 0=nonachievement).

### Twelve-Week Step Count Patterns

Step count patterns were analyzed using total weekly steps (Monday to Sunday) as a continuous variable to examine changes in step counts over the 12-week intervention period.

### Consecutive 2-Week Goal Achievement Failure Events

Consecutive 2-week goal achievement failure was defined as the first occurrence of failing to achieve the weekly 25,000-step goal for 2 consecutive weeks. This definition was selected to distinguish temporary weekly fluctuations from sustained disruptions in walking adherence. Because weekly step performance may vary due to short-term contextual factors such as weather conditions, temporary illness, or schedule changes, a single week of goal failure may reflect natural variability rather than meaningful behavioral disengagement. In contrast, consecutive failure across 2 weeks can indicate an emerging lapse in behavioral persistence and therefore provides a more stable indicator of behavioral disruption within the weekly intervention cycle used in this study.

### Statistical Analysis

#### Overview

Baseline characteristics were summarized using descriptive statistics. In accordance with CONSORT (Consolidated Standards of Reporting Trials) recommendations for RCTs, statistical significance testing between randomized groups was not performed. Baseline characteristics were compared between participants and nonparticipants after randomization to assess potential attrition bias.

#### Analysis of Weekly Goal Achievement

Generalized estimating equations (GEEs) were applied to evaluate changes in weekly goal achievement rates using logit link functions and exchangeable correlation structures to analyze the 12-week 25,000-step goal achievement status.

#### Analysis of Step Count Patterns

Linear mixed models (LMMs) were used to evaluate the 12-week step count patterns, including subject-specific random intercepts and random slopes, to account for within-individual correlations.

#### Analysis of Consecutive Goal Failure Events

Kaplan-Meier survival analysis and Cox proportional hazards models were applied to evaluate the intervention effects on consecutive 2-week goal achievement failure events.

All analyses included time, group, and time × group interactions as the main variables, with gender and age as adjustment variables. Primary analyses included a modified intention-to-treat analysis targeting 141 participants who actually participated in the intervention. Because 33 participants did not initiate the intervention and had no postbaseline outcome data, inclusion in repeated-measures analyses required at least 1 postbaseline observation. Therefore, the primary analyses followed a modified intention-to-treat approach including all participants who initiated the intervention (n=141). Sensitivity analyses using a full intention-to-treat framework including all randomized participants (n=174) with multiple imputation for missing data were additionally performed to assess robustness. Missing values were handled using multiple imputation methods. All analyses were performed using SPSS (version 28.0; IBM Corp), with statistical significance set at *P*<.05.

### Ethical Considerations

This study was approved by the Institutional Review Board of Sahmyook University (SYU 2024-02-017). All participants provided written informed consent prior to enrollment and were informed of their right to withdraw at any time without penalty. Step count data were collected through the WalkOn mobile app and deidentified prior to analysis to ensure confidentiality. Participants in both groups received identical performance-based incentives (1000-2000 points [equivalent to KRW 1000-2000] per week depending on study phase).

## Results

### Personalized Messaging Algorithm

The personalized messaging algorithm used to generate and deliver messages according to participants’ midweek step count performance is illustrated in Figure 1.

**Figure 1:**
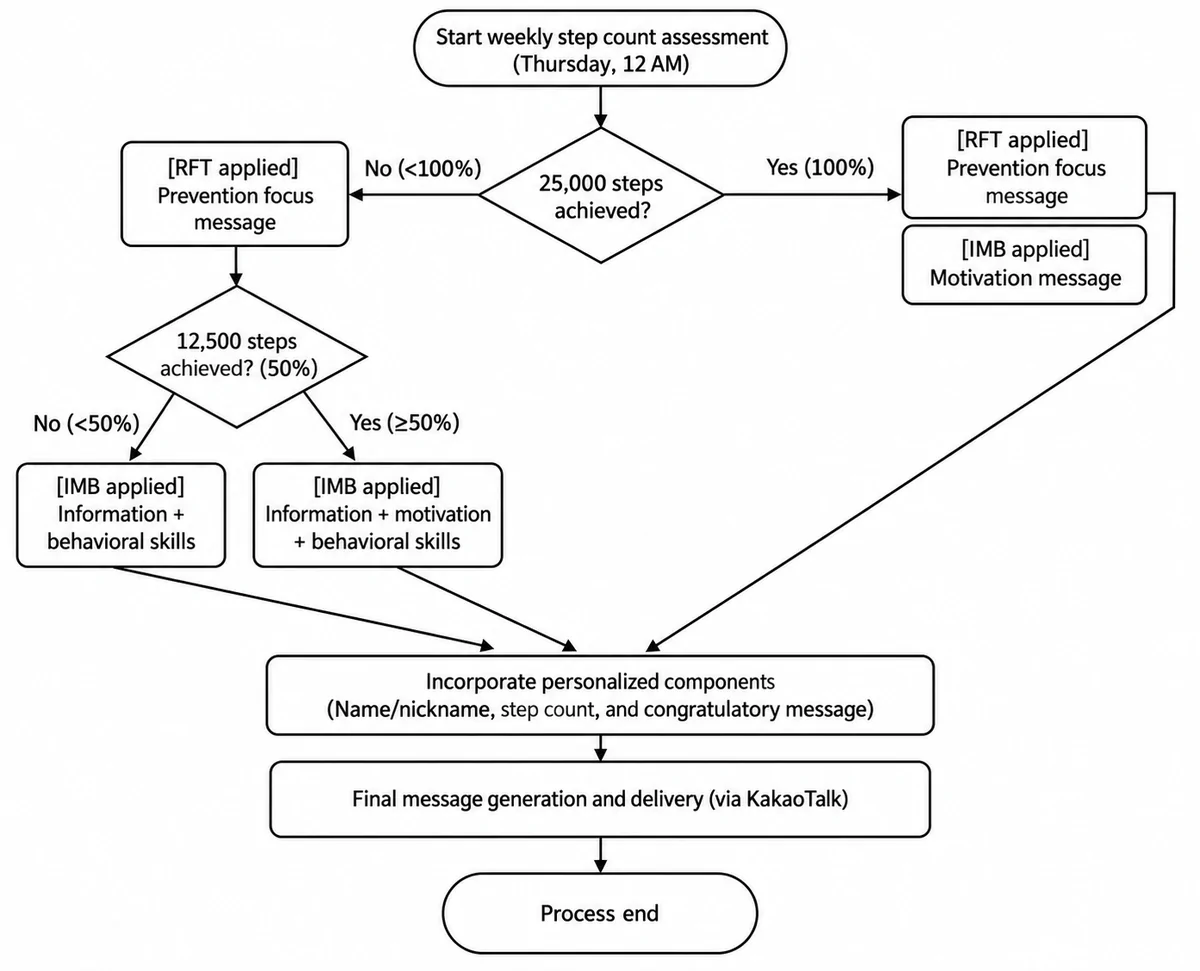
Flowchart of personalized message generation process based on weekly step count assessment. IMB: Information-Motivation-Behavioral Skills; RFT: Regulatory Focus Theory.

### Participant Characteristics and Baseline Analysis

Of the 174 randomized participants, 141 (81%) engaged in the 12-week study, including 77% (67/87) in the intervention group and 85% (74/87) in the control group. A total of 33 participants did not participate after randomization (20/87, 23%) in the intervention group and 15% (13/87) in the control group. The participant flow is presented in Figure 2.

**Figure 2:**
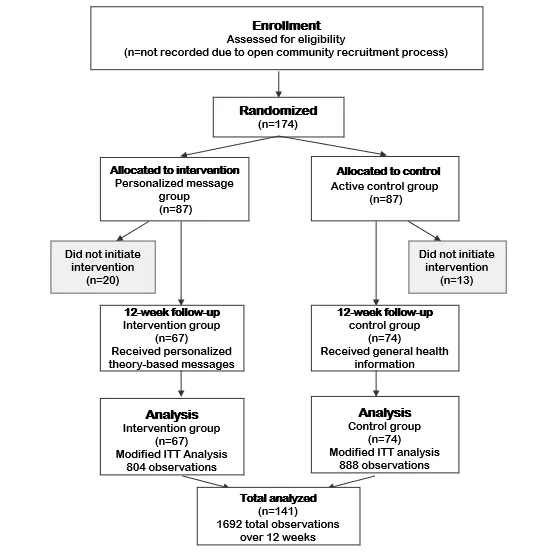
Flow of participants through the study. ITT: intention-to-treat.

Baseline characteristics of the randomized participants are presented in Table 2. In accordance with CONSORT recommendations, formal significance testing of baseline characteristics between randomized groups was not performed (CONSORT checklist is provided in Multimedia Appendix 2).

**Table 2:** Baseline characteristics of randomized participants and randomization verification. A total of 141 participants (67 in intervention; 74 in control) completed the 12-week intervention.

Variable			Total (n=174), n (%)		Intervention group (n=87), n (%)		Control group (n=87), n (%)
**Gender**							
	Men	60 (34.5)		30 (35)		30 (35)	
	Women	114 (65.5)		57 (66)		57 (66)	
**Age group (years)**							
	20-29	17 (9.8)		10 (12)		7 (8)	
	30-39	26 (14.9)		11 (13)		15 (17)	
	40-49	58 (33.3)		24 (28)		34 (39)	
	50-59	54 (31)		31 (36)		23 (26)	
	≥60	19 (10.9)		11 (13)		8 (9)	

### Population Characteristics and Selection Bias Assessment

Among the 141 participants who engaged in the intervention, borderline differences in age group distribution were observed between groups (*P*=.06), whereas no significant differences were found in gender or baseline step counts.

To assess potential attrition-related selection bias, baseline characteristics were summarized using descriptive statistics for each study group (Tables S1 and S2 in Multimedia Appendix 3).

### Intervention Effects on Weekly Goal Achievement Rates

Weekly goal achievement rates over the 12 weeks showed generally high levels in both groups; detailed week-by-week values are provided in Table S3 in Multimedia Appendix 4. The intervention group maintained relatively stable achievement rates during weeks 5 to 8, with the highest rate of 91% (61/67) observed in participants in weeks 4 to 6. The control group showed more variable patterns, with recovery in weeks 9 to 10 after midstudy declines.

GEE was applied to evaluate the effects of the personalized message interventions on weekly step count goal (25,000 steps) achievement rates over 12 weeks. Goal achievement status was set as a binary dependent variable (1=achievement; 0=nonachievement) using logit link functions and exchangeable correlation structures to account for internal correlations in repeated measurement data by subject. The model included time (week), group (intervention vs control), and time × group interaction as the main variables, analyzing 1692 observations (141 participants × 12 weeks).

GEE analysis results (Table 3) showed statistically significant changes in goal achievement rates over time (Wald *χ*^2^_11_=30.7; *P*=.001). Using week 1 as reference, most weeks did not differ significantly from week 1; however, the odds of goal achievement were significantly lower at week 12 (odds ratio [OR] 0.34, 95% CI 0.12-0.93; *P*=.04). Overall differences in goal achievement rates between intervention and control groups were not statistically significant (OR 1.12, 95% CI 0.29-4.37; *P*=.87). Additionally, time and group interaction effects were not significant (Wald *χ*^2^_11_=11.8; *P*=.38), confirming that personalized message interventions did not differentially influence goal achievement rate change patterns over time.

**Table 3:** GEE^a^ model results for weekly goal achievement rates (N=1692 observations from 141 participants).

Variable			OR^b^ (95% CI)		Wald chi-square (*df*)		**P* value*
**Time effect**							
	Week 1	Reference		—^c^		—	
	Week 2	0.81 (0.32-1.92)		0.23 (1)		.63	
	Week 3	0.58 (0.21-1.60)		1.11 (1)		.29	
	Week 4	0.80 (0.25-2.55)		0.15 (1)		.70	
	Week 5	0.79 (0.25-2.51)		0.17 (1)		.68	
	Week 6	0.57 (0.21-1.58)		1.17 (1)		.28	
	Week 7	0.56 (0.20-1.55)		1.24 (1)		.27	
	Week 8	0.65 (0.21-2.04)		0.54 (1)		.46	
	Week 9	1.62 (0.44-5.99)		0.52 (1)		.47	
	Week 10	1.59 (0.55-4.64)		0.73 (1)		.39	
	Week 11	0.55 (0.18-1.71)		1.07 (1)		.30	
	Week 12	0.34 (0.12-0.93)		4.40 (1)		.04	
Intervention group (vs control)			1.12 (0.29-4.37)		0.03 (1)		.87
Time (week)			—		30.68 (11)		.001
Group			—		1.64 (1)		.20
Time × group interaction			—		11.82 (11)		.38

^a^GEE: generalized estimating equation.

^b^OR: odds ratio.

^c^Not applicable.

### Step Count Patterns

LMM was applied to evaluate the effects of personalized message interventions on 12-week step count patterns. Step counts were set as continuous dependent variables, including subject-specific random intercepts and random slopes to account for within-individual correlations. The model included time (week), group (intervention vs control), and the time × group interaction as the main variables.

Weekly step counts over 12 weeks varied in both groups. The intervention group showed a lower overall mean weekly step count than the control group; detailed week-by-week values are presented in Table S4 in Multimedia Appendix 4. The intervention group showed a decreasing pattern from 57,217 steps in week 1 to 49,067 steps in week 12, whereas the control group showed a decreasing pattern from 60,940 steps in week 1 to 54,808 steps in week 12.

The intercept representing the estimated average weekly step count of the intervention group was 48,369 steps (SE 3524; *P*<.001, Table 4). Group effects representing between-group differences in average step counts were not significant (estimate 6184, SE 4856; *P*=.20), and step count changes over time (week) were also not significant (estimate 324, SE 642; *P*=.61). Moreover, the group × week interactions were not significant (estimate –417, SE 409; *P*=.31), confirming that personalized message interventions did not significantly influence step count patterns over 12 weeks.

**Table 4:** Linear mixed model results for weekly step count changes (N=1692 observations from 141 participants; model fit: −2 log likelihood=35,185.6; AIC^a^=35,345.6; BIC^b^=35,774.7).

Parameter	Estimate (SE)	95% CI	*P* value
Intercept	48,369 (3524)	41,437 to 55,299	<.001
Intervention group (vs control; reference: intervention)	6184 (4856)	–3369 to 15,736	.20
Week	324 (642)	–934 to 1582	.61
Group × week	–417 (409)	–1218 to 393	.31

^a^AIC: Akaike information criterion.

^b^BIC: Bayesian information criterion.

### Intervention Effects on Preventing Consecutive 2-Week Goal Achievement Failure Events

Kaplan-Meier survival analysis and Cox proportional hazards models were applied to evaluate the effects of personalized message interventions on reducing the risk of consecutive 2-week goal achievement failure events. Consecutive 2-week goal achievement failure was defined as the event of interest, and time to first occurrence was used as the survival time.

During the 12-week observation period, 37% (27/74) of participants in the control group and 34% (23/67) of participants in the intervention group experienced consecutive 2-week goal achievement failure events. The average time to first consecutive failure was 4.7 weeks in the control group and 4.9 weeks in the intervention group. Detailed event frequencies and survival statistics are presented in Tables S5 and S6 in Multimedia Appendix 5.

Kaplan-Meier survival analysis demonstrated a statistically significant difference in time to consecutive 2-week goal achievement failure between groups (log-rank *χ*^2^_1_=9.9; *P*=.002; Figure 3). By the end of the 12-week follow-up, more than half of the participants in both groups had not experienced consecutive failure events; therefore, the median survival time was not reached. The intervention group showed longer survival without consecutive failure compared with the control group, indicating improved resilience against repeated goal failure.

**Figure 3:**
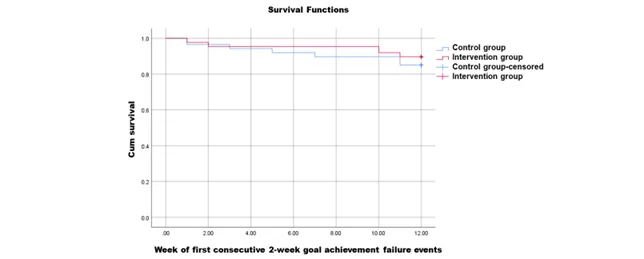
Kaplan-Meier survival curves for consecutive 2-week goal achievement failure.

The Cox proportional hazards model analysis results (Table 5) showed that the intervention group had a significantly reduced risk of consecutive 2-week goal achievement failure events compared with the control group (hazard ratio [HR] 0.68, 95% CI 0.53-0.87; *P*=.002). In models adjusted for gender and age group, the intervention group still had a significantly lower risk of consecutive 2-week goal achievement failure events than the control group (HR 0.67, 95% CI 0.52-0.86; *P*=.002), representing an approximately 32.9% risk reduction.

**Table 5:** Cox proportional hazards model results for consecutive 2-week goal achievement failure events.

Variable		Unadjusted model, HR^a^ (95% CI)	Adjusted Model^b^, HR (95% CI)	*P* value
Intervention group (vs control)		0.68 (0.53-0.87)	0.67 (0.52-0.86)	.002
Women (vs men)		—^c^	0.43 (0.33–0.55)	<.001
**Age group (years; reference: 20-29)**				
	30-39	—	1.46 (0.76–2.83)	.26
	40-49	—	1.48 (0.80–2.73)	.21
	50-59	—	2.00 (1.09–3.67)	.03
	≥60	—	1.62 (0.83–3.17)	.16

^a^HR: hazard ratio.

^b^Adjusted for gender and age group.

^c^Not applicable.

Among the adjustment variables, women had a significantly lower risk of consecutive 2-week goal achievement failure events than men (HR 0.43, 95% CI 0.33-0.55; *P*<.001), and by age group, participants aged 50-59 years had approximately twice the risk of consecutive 2-week goal achievement failure events compared with those aged 20-29 years (HR 2.00, 95% CI 1.09-3.67; *P*=.03).

Kaplan-Meier survival curves also showed that the intervention group maintained a higher survival probability for consecutive 2-week goal achievement failure events than the control group throughout the study period (Figure 2), consistent with the Cox proportional hazards model results.

### Sensitivity Analyses

Sensitivity analyses including all randomized participants (n=174) yielded results consistent in direction with the primary analysis. The intervention group showed a lower risk of consecutive 2-week goal achievement failure (HR 0.67, 95% CI 0.29-1.58), although this was not statistically significant (*P*=.37).

## Discussion

### Interpretation of Main Results

This RCT found that personalized theory-based messages did not significantly improve overall weekly step goal achievement rates or step count patterns over 12 weeks but significantly reduced the risk of consecutive 2-week goal achievement failures (HR 0.67, 95% CI 0.52-0.86; *P*=.002). These findings suggest that personalized messaging may enhance behavioral resilience by preventing repeated goal failures, even if it does not substantially increase overall performance levels.

These results are particularly noteworthy in the context of the study design. The control group in this study was not a simple comparison group but received substantial active intervention. The control group participants received the same progressive incentive system as the intervention group (weeks 1-4: 1000 points [KRW 1000]; weeks 5-8: 1500 points [KRW 1500]; weeks 9-12: 2000 points [KRW 2000]) and continuously received general health information through weekly walking and health promotion card news. They also experienced social comparison and competitive motivation through real-time participant step count comparison features via the WalkOn app and received the same weekly 25,000-step goal as the intervention group.

The significant effect of personalized messages on consecutive failure prevention, even in this strong control group intervention environment, suggests the unique value of personalization. Particularly, despite both groups maintaining high goal achievement rates (intervention group: 87%, control group: 87%), the approximately 33% reduction in consecutive 2-week goal achievement failure events in the intervention group demonstrates that personalized messages play a special role in improving “failure resilience” [43,44].

Compared with existing research, the results of this study have a deeper meaning. While most digital walking intervention studies have reported only short-term step count increases using minimal intervention control groups [11,12], this study verified the pure effects of personalized messages through an active control group design. This represents an important contribution by clearly identifying the actual effects of personalized interventions while addressing the methodological limitations of existing research. Consecutive failure prevention effects suggest that personalized feedback positively influences behavioral sustainability [45-47], and confirming these results under rigorous control conditions demonstrates the unique contribution of this study.

Recent evidence has also demonstrated the effectiveness of personalized digital messaging for increasing physical activity [48]. For example, an RCT using a reinforcement learning–based adaptive messaging system reported that personalized messages tailored to participants’ behavioral data significantly increased daily step counts compared with nonpersonalized messaging approaches. These findings support the broader evidence that personalization strategies can enhance behavioral engagement and physical activity in digital health interventions.

However, unlike previous studies that primarily focused on increasing overall step counts, the present study evaluated whether personalized theory-based messages could prevent repeated weekly goal achievement failure. While our intervention did not significantly increase mean step counts compared with the control condition, it significantly reduced the risk of consecutive goal achievement failure. This suggests that personalized feedback may play a particularly important role in sustaining behavioral adherence and preventing disengagement rather than simply increasing activity volume.

The goal achievement rate increase pattern observed during the middle period (weeks 4-8) is particularly interesting. This suggests that personalized messages may have acted as protective factors when the novelty effects were diminishing [49]. The maximum 6 percentage point achievement rate difference at week 6 demonstrates that personalized messages are particularly effective during the “dropout risk period” of intervention participation [49,50]. This emphasizes the need for individualized motivational support, which cannot be sufficiently provided through general health information or social comparison alone [51].

### Theoretical Contribution

This study contributes theoretically by systematically implementing differentiated messaging strategies according to individual performance levels by applying a theoretical framework that integrates the IMB model and RFT. While existing research primarily relied on single theories [52-54], this study attempted an adaptive approach that comprehensively addresses information, motivation, and behavioral skills while dynamically adjusting prevention- or promotion-focused messages according to individual performance changes.

The preventive effects on consecutive 2-week goal achievement failure events align with the theoretical predictions that RFT’s prevention focus supports behavioral sustainability through loss avoidance motivation [32]. Particularly, prevention-focused messages provided to participants with fewer than 12,500 steps may have effectively prevented goal abandonment. This demonstrates the importance of theory-based approaches that consider individual motivational orientations beyond simple performance feedback in personalized messages [53,55,56].

### Practical Implications

The practical implications of this study are as follows. First, the finding that personalized messages are effective in preventing consecutive failures provides important insights for developing user retention strategies in the mobile health care industry. Considering the high dropout rates (over 40%) observed in existing wearable devices and smartphone applications, according to a meta-research study [57], the responsive messaging system developed in this study can be used as a cost-effective solution for improving user engagement.

Second, the differential effects of gender and age group (women: HR 0.43, 95% CI 0.33-0.55; age group 50-59 years: HR 2.00, 95% CI 1.09-3.67) emphasize the importance of personalized approaches that consider demographic characteristics in personalized intervention design [58,59]. This study provides evidence for establishing differentiated strategies for target populations in national-level physical activity promotion policies [60].

Third, the weekly performance-based message reflection system developed in this study can be extended to other areas of health behavior change, such as chronic disease management, smoking cessation, and dietary improvement. Particularly, it can provide important evidence for the systematic application of behavior change theories and personalized algorithm designs in digital therapeutics development [61].

### Study Limitations

This study has several limitations. First, although an a priori sample size calculation was conducted, the achieved sample size was smaller than the original recruitment target. As a result, statistical power to detect small between-group differences may have been limited, and nonsignificant findings for overall goal achievement and step count patterns should be interpreted cautiously. Therefore, this trial should not be interpreted as a fully powered confirmatory trial for detecting small between-group differences. Second, the relatively short 12-week follow-up period limited the ability to assess long-term maintenance of behavior change. Given the sharp decline in engagement after 6 months reported in previous studies [62], longer-term follow-up trials are warranted. Third, higher nonparticipation rates were observed in the intervention group (24.1% vs 14.9%). This difference may reflect the additional engagement burden associated with personalized messaging or the psychological pressure of continuous performance monitoring [63]. Although no significant baseline differences were observed between participants and nonparticipants, residual attrition-related bias due to postrandomization nonparticipation cannot be excluded. Fourth, participants were recruited from a single urban community in Mapo-gu, Seoul, which may limit generalizability to other regional or cultural contexts. Moreover, the trial included progressive monetary incentives and weekly researcher monitoring, conditions that may not be replicated in routine real-world implementation. Fifth, step counts were measured using built-in smartphone accelerometers, which may not capture all forms of physical activity and may vary in accuracy across devices. Participants were not blinded to group allocation, which may have introduced performance or expectation bias, a common limitation in eHealth trials. In addition, although primary outcomes were prespecified, the analysis of multiple outcomes raises the possibility of an inflated type I error.

### Future Research Directions

Based on the results of this study, we propose the following follow-up research. First, long-term follow-up studies of 6 months or more should evaluate the sustained effects of personalized messages and their impact on habit formation. Second, research is needed to develop machine learning-based adaptive messaging systems that learn individual real-time response patterns and implement more sophisticated personalization. Third, generalizability should be verified through multisite RCTs targeting diverse populations (rural areas, older adults, and patients with chronic diseases). Fourth, research on optimizing message delivery methods (push notifications, chatbots, and voice messages) and message transmission frequency is required. Finally, research is necessary to comprehensively evaluate message acceptability, fatigue, privacy concerns, and so on through user experience research to increase implementation feasibility in actual clinical settings.

### Conclusion

This study empirically evaluated the effects of personalized message interventions that integrate the IMB model and RFT on walking behavior maintenance. Although these interventions did not significantly affect the overall increase in step counts, they were confirmed to be effective in preventing consecutive goal achievement failures. Personalized tailored messages did not significantly affect the overall increase in step counts but had significant effects in preventing consecutive 2-week goal achievement failures. This demonstrates that personalized messages are important variables that enhance behavioral sustainability rather than simple performance improvement. This study provides important evidence for establishing user retention strategies in digital health care intervention design, showing that personalized feedback can contribute to improving user resilience and engagement sustainability. Performance-based personalized messaging strategies can be applied to various health behavior change areas for developing digital therapeutics or healthy lifestyle habits in the future. Future research should assess the long-term sustainability of personalized messaging and determine how these findings can support the refinement of adaptive digital health personalization systems.

## Data Availability

The data supporting the findings of this study are available from the corresponding author upon reasonable request.

## Appendix

Multimedia Appendix 1 Examples of personalized messages sent by performance level.

Multimedia Appendix 2 CONSORT checklist.

Multimedia Appendix 3 Participant/nonparticipant comparison and attrition bias assessment.

Multimedia Appendix 4 Detailed weekly step count data and weekly mean step counts.

Multimedia Appendix 5 Additional survival analysis details.

## Abbreviations

BCT: behavior change technique
CONSORT: Consolidated Standards of Reporting Trials
CRIS: Clinical Research Information Service
GEE: generalized estimating equation
HR: hazard ratio
IMB: Information-Motivation-Behavioral Skills
LMM: linear mixed models
MET: metabolic equivalent
OR: odds ratio
RCT: randomized controlled trial
RFT: regulatory focus theory
WHO: World Health Organization
