# Supplementary material for: Personalized Mobile App–Based Messaging and Step Tracking for Improving Walking Behavior in Adults: Randomized Controlled Trial
Source: JMIR Mhealth Uhealth. 2026 Sep 17;14:e84430. doi: 10.2196/84430 (PMC13631492; doi:10.2196/84430)
Supplement: Supplementary file 1 [file mhealth_v14i1e84430_app2.pdf]

CONSORT-EHEALTH (V 1.6.1) - Submission/Publication Form

The CONSORT-EHEALTH checklist is intended for authors of randomized trials evaluating web-based and Internet-based applications/interventions, including mobile interventions, electronic games (incl multiplayer games), social media, certain telehealth applications, and other interactive and/or networked electronic applications. Some of the items (e.g. all subitems under item 5 - description of the intervention) may also be applicable for other study designs.

The goal of the CONSORT EHEALTH checklist and guideline is to be
a) a guide for reporting for authors of RCTs,
b) to form a basis for appraisal of an ehealth trial (in terms of validity)

CONSORT-EHEALTH items/subitems are MANDATORY reporting items for studies published in the Journal of Medical Internet Research and other journals / scientific societies endorsing the checklist.

Items numbered 1., 2., 3., 4a., 4b etc are original CONSORT or CONSORT-NPT (non-pharmacologic treatment) items.
Items with Roman numerals (i., ii, iii, iv etc.) are CONSORT-EHEALTH extensions/clarifications.

As the CONSORT-EHEALTH checklist is still considered in a formative stage, we would ask that you also RATE ON A SCALE OF 1-5 how important/useful you feel each item is FOR THE PURPOSE OF THE CHECKLIST and reporting guideline (optional).

Mandatory reporting items are marked with a red *.
In the textboxes, either copy & paste the relevant sections from your manuscript into this form - please include any quotes from your manuscript in QUOTATION MARKS,
or answer directly by providing additional information not in the manuscript, or elaborating on why the item was not relevant for this study.

YOUR ANSWERS WILL BE PUBLISHED AS A SUPPLEMENTARY FILE TO YOUR PUBLICATION IN JMIR AND ARE CONSIDERED PART OF YOUR PUBLICATION (IF ACCEPTED).
Please fill in these questions diligently. Information will not be copyedited, so please use proper spelling and grammar, use correct capitalization, and avoid abbreviations.

DO NOT FORGET TO SAVE AS PDF _AND_ CLICK THE SUBMIT BUTTON SO YOUR ANSWERS ARE IN OUR DATABASE !!!

Citation Suggestion (if you append the pdf as Appendix we suggest to cite this paper in the caption):
Eysenbach G, CONSORT-EHEALTH Group

응답이 너무 큽니다. 일부 응답을 짧게 줄여주세요.

J Med Internet Res 2011;13(4):e126

URL: http://www.jmir.org/2011/4/e126/
doi: 10.2196/jmir.1923
PMID: 22209829

jangsarang@gmail.com 계정 전환

비공개

저장하려면 다시 제출하세요

* 표시는 필수 질문임

Your name *

First Last

Sarang

Primary Affiliation (short), City, Country *

University of Toronto, Toronto, Canada

Sahmyook University, Seoul, Korea

Your e-mail address *

abc@gmail.com

srjang@syu.ac.kr

Title of your manuscript *

Provide the (draft) title of your manuscript.

Digital Health Intervention Using Personalized Messages and Step Tracking: A Randomized Controlled Trial to Prevent Walking Goal Failure

응답이 너무 큽니다. 일부 응답을 짧게 줄여주세요.

Name of your App/Software/Intervention *

If there is a short and a long/alternate name, write the short name first and add the long name in brackets.

Kobak-ttubak (A personalized mobile walking i

Evaluated Version (if any)

e.g. "V1", "Release 2017-03-01", "Version 2.0.27913"

내 답변

Language(s) *

What language is the intervention/app in? If multiple languages are available, separate by comma (e.g. "English, French")

Korean

URL of your Intervention Website or App

e.g. a direct link to the mobile app on app in appstore (itunes, Google Play), or URL of the website. If the intervention is a DVD or hardware, you can also link to an Amazon page.

https://play.google.com/store/apps/details?id=com.swallaby.walkon, http://pf.kakao.com/_x

URL of an image/screenshot (optional)

내 답변

응답이 너무 큽니다. 일부 응답을 짧게 줄여주세요.

Accessibility *

Can an enduser access the intervention presently?

- ○ access is free and open
- ◉ access only for special usergroups, not open
- ○ access is open to everyone, but requires payment/subscription/in-app purchases
- ○ app/intervention no longer accessible
- ○ 기타:

Primary Medical Indication/Disease/Condition *

e.g. "Stress", "Diabetes", or define the target group in brackets after the condition, e.g. "Autism (Parents of children with)", "Alzheimers (Informal Caregivers of)"

Physical Activity Promotion (Community-dwell

Primary Outcomes measured in trial *

comma-separated list of primary outcomes reported in the trial

Physical inactivity (Community-dwelling adults

Secondary/other outcomes

Are there any other outcomes the intervention is expected to affect?

Step count (weekly average steps)

Walking consistency (e.g., consecutive weekly goal achievement)

WHO physical activity guideline adherence

Motivation for walking

Stage of change (behavioral readiness for walking)

응답이 너무 큽니다. 일부 응답을 짧게 줄여주세요.

Recommended "Dose" *

What do the instructions for users say on how often the app should be used?

- (selected) Approximately Daily
- Approximately Weekly
- Approximately Monthly
- Approximately Yearly
- "as needed"
- 기타:

Approx. Percentage of Users (starters) still using the app as recommended after 3 months *

- unknown / not evaluated
- 0-10%
- 11-20%
- 21-30%
- 31-40%
- 41-50%
- 51-60%
- 61-70%
- (selected) 71%-80%
- 81-90%
- 91-100%
- 기타:

응답이 너무 큽니다. 일부 응답을 짧게 줄여주세요.

Overall, was the app/intervention effective? *

- ○ yes: all primary outcomes were significantly better in intervention group vs control
- ◉ partly: SOME primary outcomes were significantly better in intervention group vs control
- ○ no statistically significant difference between control and intervention
- ○ potentially harmful: control was significantly better than intervention in one or more outcomes
- ○ inconclusive: more research is needed
- ○ 기타:

Article Preparation Status/Stage *

At which stage in your article preparation are you currently (at the time you fill in this form)

- ○ not submitted yet - in early draft status
- ○ not submitted yet - in late draft status, just before submission
- ○ submitted to a journal but not reviewed yet
- ◉ submitted to a journal and after receiving initial reviewer comments
- ○ submitted to a journal and accepted, but not published yet
- ○ published
- ○ 기타:

응답이 너무 큽니다. 일부 응답을 짧게 줄여주세요.

Journal *

If you already know where you will submit this paper (or if it is already submitted), please provide the journal name (if it is not JMIR, provide the journal name under "other")

- ○ not submitted yet / unclear where I will submit this
- ○ Journal of Medical Internet Research (JMIR)
- ○ JMIR mHealth and UHealth
- ○ JMIR Serious Games
- ○ JMIR Mental Health
- ◉ JMIR Public Health
- ○ JMIR Formative Research
- ○ Other JMIR sister journal
- ○ 기타:

Is this a full powered effectiveness trial or a pilot/feasibility trial? *

- ○ Pilot/feasibility
- ◉ Fully powered

Manuscript tracking number *

If this is a JMIR submission, please provide the manuscript tracking number under "other" (The ms tracking number can be found in the submission acknowledgement email, or when you login as author in JMIR. If the paper is already published in JMIR, then the ms tracking number is the four-digit number at the end of the DOI, to be found at the bottom of each published article in JMIR)

- ○ no ms number (yet) / not (yet) submitted to / published in JMIR
- ◉ 기타: 84430-1250823-1-SM

응답이 너무 큽니다. 일부 응답을 짧게 줄여주세요.

TITLE AND ABSTRACT

1a) TITLE: Identification as a randomized trial in the title

1a) Does your paper address CONSORT item 1a? *

I.e does the title contain the phrase "Randomized Controlled Trial"? (if not, explain the reason under "other")

- (●) yes
- ( ) 기타:

1a-i) Identify the mode of delivery in the title

Identify the mode of delivery. Preferably use “web-based” and/or “mobile” and/or “electronic game” in the title. Avoid ambiguous terms like “online”, “virtual”, “interactive”. Use “Internet-based” only if Intervention includes non-web-based Internet components (e.g. email), use “computer-based” or “electronic” only if offline products are used. Use “virtual” only in the context of “virtual reality” (3-D worlds). Use “online” only in the context of “online support groups”. Complement or substitute product names with broader terms for the class of products (such as “mobile” or “smart phone” instead of “iphone”), especially if the application runs on different platforms.

	1	2	3	4	5	
subitem not at all important	( )	( )	( )	( )	(●)	essential

선택해제

응답이 너무 큽니다. 일부 응답을 짧게 줄여주세요.

Does your paper address subitem 1a-i? *

Copy and paste relevant sections from manuscript title (include quotes in quotation marks "like this" to indicate direct quotes from your manuscript), or elaborate on this item by providing additional information not in the ms, or briefly explain why the item is not applicable/relevant for your study

Yes, our paper addresses subitem 1a-i.
The intervention was delivered through the WalkOn mobile app, which tracked steps and sent weekly personalized messages.
Although the original title did not state “mobile,” a title revision to include “Mobile App–Based” has been requested for clearer reporting and guideline compliance.

1a-ii) Non-web-based components or important co-interventions in title

Mention non-web-based components or important co-interventions in title, if any (e.g., “with telephone support”).

	1	2	3	4	5	
subitem not at all important	○	◉	○	○	○	essential

선택해제

Does your paper address subitem 1a-ii?

Copy and paste relevant sections from manuscript title (include quotes in quotation marks "like this" to indicate direct quotes from your manuscript), or elaborate on this item by providing additional information not in the ms, or briefly explain why the item is not applicable/relevant for your study

내 답변

응답이 너무 큽니다. 일부 응답을 짧게 줄여주세요.

1a-iii) Primary condition or target group in the title

Mention primary condition or target group in the title, if any (e.g., “for children with Type I Diabetes”) Example: A Web-based and Mobile Intervention with Telephone Support for Children with Type I Diabetes: Randomized Controlled Trial

	1	2	3	4	5	
subitem not at all important	○	◉	○	○	○	essential

선택해제

Does your paper address subitem 1a-iii? *

Copy and paste relevant sections from manuscript title (include quotes in quotation marks "like this" to indicate direct quotes from your manuscript), or elaborate on this item by providing additional information not in the ms, or briefly explain why the item is not applicable/relevant for your study

The title does not name a specific condition or target group because the intervention targeted general community-dwelling adults to promote walking, not to treat a particular disease.
This omission was intentional to reflect the broad, non-clinical focus of the study.

1b) ABSTRACT: Structured summary of trial design, methods, results, and conclusions

NPT extension: Description of experimental treatment, comparator, care providers, centers, and blinding status.

응답이 너무 큽니다. 일부 응답을 짧게 줄여주세요.

1b-i) Key features/functionalities/components of the intervention and comparator in the METHODS section of the ABSTRACT

Mention key features/functionalities/components of the intervention and comparator in the abstract. If possible, also mention theories and principles used for designing the site. Keep in mind the needs of systematic reviewers and indexers by including important synonyms. (Note: Only report in the abstract what the main paper is reporting. If this information is missing from the main body of text, consider adding it)

	1	2	3	4	5	
subitem not at all important	○	○	○	○	◉	essential

선택해제

Does your paper address subitem 1b-i? *

Copy and paste relevant sections from the manuscript abstract (include quotes in quotation marks "like this" to indicate direct quotes from your manuscript), or elaborate on this item by providing additional information not in the ms, or briefly explain why the item is not applicable/relevant for your study

Yes. The Methods section of the abstract clearly explains both the intervention and comparator and cites the theoretical frameworks.
The intervention delivered personalized mobile app messages based on mid-week step counts, guided by the Information-Motivation-Behavioral Skills model and Regulatory Focus Theory, while the control group received general health information with identical incentives but no personalization.

1b-ii) Level of human involvement in the METHODS section of the ABSTRACT

Clarify the level of human involvement in the abstract, e.g., use phrases like “fully automated” vs. “therapist/nurse/care provider/physician-assisted” (mention number and expertise of providers involved, if any). (Note: Only report in the abstract what the main paper is reporting. If this information is missing from the main body of text, consider adding it)

	1	2	3	4	5	
subitem not at all important	○	○	○	○	◉	essential

선택해제

응답이 너무 큽니다. 일부 응답을 짧게 줄여주세요.

Does your paper address subitem 1b-ii?

Copy and paste relevant sections from the manuscript abstract (include quotes in quotation marks "like this" to indicate direct quotes from your manuscript), or elaborate on this item by providing additional information not in the ms, or briefly explain why the item is not applicable/relevant for your study

Yes.
From the revised abstract:
"…whereas the intervention group received weekly personalized messages provided by research staff according to midweek step count performance."

1b-iii) Open vs. closed, web-based (self-assessment) vs. face-to-face assessments in the METHODS section of the ABSTRACT

Mention how participants were recruited (online vs. offline), e.g., from an open access website or from a clinic or a closed online user group (closed usergroup trial), and clarify if this was a purely web-based trial, or there were face-to-face components (as part of the intervention or for assessment). Clearly say if outcomes were self-assessed through questionnaires (as common in web-based trials). Note: In traditional offline trials, an open trial (open-label trial) is a type of clinical trial in which both the researchers and participants know which treatment is being administered. To avoid confusion, use "blinded" or "unblinded" to indicated the level of blinding instead of "open", as "open" in web-based trials usually refers to "open access" (i.e. participants can self-enrol). (Note: Only report in the abstract what the main paper is reporting. If this information is missing from the main body of text, consider adding it)

	1	2	3	4	5	
subitem not at all important	○	○	○	○	◉	essential

선택해제

응답이 너무 큽니다. 일부 응답을 짧게 줄여주세요.

Does your paper address subitem 1b-iii?

Copy and paste relevant sections from the manuscript abstract (include quotes in quotation marks "like this" to indicate direct quotes from your manuscript), or elaborate on this item by providing additional information not in the ms, or briefly explain why the item is not applicable/relevant for your study

Yes. The revised abstract Methods clearly describes recruitment and enrollment procedures. Participants were recruited offline via QR-code community ads, enrolled through an online sign-up form (closed recruitment), and screened online by research staff.
All intervention delivery and outcome assessments were conducted entirely through the WalkOn mobile app. These details fulfill CONSORT subitem 1b-iii by specifying recruitment method, closed enrollment, and fully web-based intervention and assessment.

1b-iv) RESULTS section in abstract must contain use data

Report number of participants enrolled/assessed in each group, the use/uptake of the intervention (e.g., attrition/adherence metrics, use over time, number of logins etc.), in addition to primary/secondary outcomes. (Note: Only report in the abstract what the main paper is reporting. If this information is missing from the main body of text, consider adding it)

	1	2	3	4	5	
subitem not at all important	○	○	○	○	◉	essential

선택해제

응답이 너무 큽니다. 일부 응답을 짧게 줄여주세요.

Does your paper address subitem 1b-iv?

Copy and paste relevant sections from the manuscript abstract (include quotes in quotation marks "like this" to indicate direct quotes from your manuscript), or elaborate on this item by providing additional information not in the ms, or briefly explain why the item is not applicable/relevant for your study

The revised abstract now includes key participation and outcome data. Of 174 randomized participants, 141 (81.0%) took part in the intervention (67 intervention, 74 control) and 33 dropped out. Average 12-week goal achievement rates were similar between groups (intervention 87.2% vs control 86.6%), and weekly step count patterns showed no significant group difference. However, the intervention group had a 33% lower risk of consecutive 2-week goal-achievement failure (HR 0.67, 95% CI 0.52–0.86). These details meet CONSORT-eHEALTH requirements for reporting enrollment, uptake, and primary outcomes in the abstract.

1b-v) CONCLUSIONS/DISCUSSION in abstract for negative trials

Conclusions/Discussions in abstract for negative trials: Discuss the primary outcome - if the trial is negative (primary outcome not changed), and the intervention was not used, discuss whether negative results are attributable to lack of uptake and discuss reasons. (Note: Only report in the abstract what the main paper is reporting. If this information is missing from the main body of text, consider adding it)

	1	2	3	4	5	
subitem not at all important	○	○	○	○	◉	essential

선택해제

Does your paper address subitem 1b-v?

Copy and paste relevant sections from the manuscript abstract (include quotes in quotation marks "like this" to indicate direct quotes from your manuscript), or elaborate on this item by providing additional information not in the ms, or briefly explain why the item is not applicable/relevant for your study

The revised abstract clearly reports a negative primary outcome: personalized message interventions did not significantly increase overall step counts but did reduce consecutive 2-week goal-failure events. This fulfills CONSORT-eHEALTH requirements by explicitly stating the nonsignificant main result while highlighting a secondary positive effect.

응답이 너무 큽니다. 일부 응답을 짧게 줄여주세요.

INTRODUCTION

2a) In INTRODUCTION: Scientific background and explanation of rationale

2a-i) Problem and the type of system/solution

Describe the problem and the type of system/solution that is object of the study: intended as stand-alone intervention vs. incorporated in broader health care program? Intended for a particular patient population? Goals of the intervention, e.g., being more cost-effective to other interventions, replace or complement other solutions? (Note: Details about the intervention are provided in “Methods” under 5)

	1	2	3	4	5	
subitem not at all important	○	○	○	○	◉	essential

선택해제

Does your paper address subitem 2a-i? *

Copy and paste relevant sections from the manuscript (include quotes in quotation marks "like this" to indicate direct quotes from your manuscript), or elaborate on this item by providing additional information not in the ms, or briefly explain why the item is not applicable/relevant for your study

The manuscript addresses subitem 2a-i by outlining the problem (physical inactivity and poor sustainability of digital step-count programs), the solution (WalkOn app with personalized KakaoTalk messages), the target group (community-dwelling Korean adults able to walk and use smartphones), and the goal (enhancing long-term walking and preventing consecutive goal failures using an IMB + RFT–based messaging system).

응답이 너무 큽니다. 일부 응답을 짧게 줄여주세요.

2a-ii) Scientific background, rationale: What is known about the (type of) system

Scientific background, rationale: What is known about the (type of) system that is the object of the study (be sure to discuss the use of similar systems for other conditions/diagnoses, if appropiate), motivation for the study, i.e. what are the reasons for and what is the context for this specific study, from which stakeholder viewpoint is the study performed, potential impact of findings [2]. Briefly justify the choice of the comparator.

	1	2	3	4	5	
subitem not at all important	○	○	○	○	◉	essential

선택해제

Does your paper address subitem 2a-ii? *

Copy and paste relevant sections from the manuscript (include quotes in quotation marks "like this" to indicate direct quotes from your manuscript), or elaborate on this item by providing additional information not in the ms, or briefly explain why the item is not applicable/relevant for your study

The Introduction explains that digital step-counting apps boost activity short term but rarely sustain long-term walking. To address this, the study applies a theory-based personalized messaging system integrating IMB and RFT to improve adherence and prevent goal failures. Focusing on community-dwelling adults, it aims to inform sustainable digital health strategies and national activity policies, using an active control group to isolate the effect of personalized messaging.

2b) In INTRODUCTION: Specific objectives or hypotheses

Does your paper address CONSORT subitem 2b? *

Copy and paste relevant sections from the manuscript (include quotes in quotation marks "like this" to indicate direct quotes from your manuscript), or elaborate on this item by providing additional information not in the ms, or briefly explain why the item is not applicable/relevant for your study

Yes. This information was already included in the original manuscript and remains unchanged in the revised version that will be submitted to the editorial office.

응답이 너무 큽니다. 일부 응답을 짧게 줄여주세요.

METHODS

3a) Description of trial design (such as parallel, factorial) including allocation ratio

Does your paper address CONSORT subitem 3a? *

Copy and paste relevant sections from the manuscript (include quotes in quotation marks "like this" to indicate direct quotes from your manuscript), or elaborate on this item by providing additional information not in the ms, or briefly explain why the item is not applicable/relevant for your study

Yes. The trial design is clearly described and remains unchanged in the revised version.

3b) Important changes to methods after trial commencement (such as eligibility criteria), with reasons

Does your paper address CONSORT subitem 3b? *

Copy and paste relevant sections from the manuscript (include quotes in quotation marks "like this" to indicate direct quotes from your manuscript), or elaborate on this item by providing additional information not in the ms, or briefly explain why the item is not applicable/relevant for your study

The manuscript reports a key procedural change: retrospective trial registration. Although IRB approval was obtained (SYU 2024-02-017), the trial was registered with CRIS (KCT0010966) only after it began because preregistration was not recognized as necessary in Korea at the time. The authors promptly completed registration and confirmed that all research procedures matched the IRB-approved protocol, with no other methodological changes after trial initiation.

응답이 너무 큽니다. 일부 응답을 짧게 줄여주세요.

3b-i) Bug fixes, Downtimes, Content Changes

Bug fixes, Downtimes, Content Changes: ehealth systems are often dynamic systems. A description of changes to methods therefore also includes important changes made on the intervention or comparator during the trial (e.g., major bug fixes or changes in the functionality or content) (5-iii) and other "unexpected events" that may have influenced study design such as staff changes, system failures/downtimes, etc. [2].

	1	2	3	4	5	
subitem not at all important	○	○	○	○	◉	essential

선택해제

Does your paper address subitem 3b-i?

Copy and paste relevant sections from the manuscript (include quotes in quotation marks "like this" to indicate direct quotes from your manuscript), or elaborate on this item by providing additional information not in the ms, or briefly explain why the item is not applicable/relevant for your study

This subitem is applicable, but no relevant changes occurred during the trial. As stated in the manuscript, "This study was a randomized controlled trial conducted for 12 weeks … to evaluate the effects of personalized health message interventions on walking behavior in Korean adults." The WalkOn mobile application and the KakaoTalk messaging system were used throughout the 12-week trial without any major bug fixes, downtimes, or content changes that could have influenced the study design or outcomes.

4a) Eligibility criteria for participants

Does your paper address CONSORT subitem 4a? *

Copy and paste relevant sections from the manuscript (include quotes in quotation marks "like this" to indicate direct quotes from your manuscript), or elaborate on this item by providing additional information not in the ms, or briefly explain why the item is not applicable/relevant for your study

The revised manuscript fully meets CONSORT 4a by detailing participant criteria.

응답이 너무 큽니다. 일부 응답을 짧게 줄여주세요.

4a-i) Computer / Internet literacy

Computer / Internet literacy is often an implicit "de facto" eligibility criterion - this should be explicitly clarified.

	1	2	3	4	5	
subitem not at all important	○	○	○	○	◉	essential

선택해제

Does your paper address subitem 4a-i?

Copy and paste relevant sections from the manuscript (include quotes in quotation marks "like this" to indicate direct quotes from your manuscript), or elaborate on this item by providing additional information not in the ms, or briefly explain why the item is not applicable/relevant for your study

The manuscript addresses this subitem by noting that owning a smartphone capable of installing the app ensured a basic level of digital literacy. Adults ≥19 years in Mapo-gu were recruited via community ads and screened face-to-face. Inclusion required smartphone ownership, app installation ability, independent walking, and signed consent, while no additional digital literacy assessment beyond smartphone use was conducted.

4a-ii) Open vs. closed, web-based vs. face-to-face assessments:

Open vs. closed, web-based vs. face-to-face assessments: Mention how participants were recruited (online vs. offline), e.g., from an open access website or from a clinic, and clarify if this was a purely web-based trial, or there were face-to-face components (as part of the intervention or for assessment), i.e., to what degree got the study team to know the participant. In online-only trials, clarify if participants were quasi-anonymous and whether having multiple identities was possible or whether technical or logistical measures (e.g., cookies, email confirmation, phone calls) were used to detect/prevent these.

	1	2	3	4	5	
subitem not at all important	○	○	○	○	◉	essential

선택해제

응답이 너무 큽니다. 일부 응답을 짧게 줄여주세요.

Does your paper address subitem 4a-ii? *

Copy and paste relevant sections from the manuscript (include quotes in quotation marks "like this" to indicate direct quotes from your manuscript), or elaborate on this item by providing additional information not in the ms, or briefly explain why the item is not applicable/relevant for your study

The revised manuscript specifies that recruitment was conducted offline via community posters and bulletin boards containing a QR code linked to an online sign-up form (closed recruitment).

4a-iii) Information giving during recruitment

Information given during recruitment. Specify how participants were briefed for recruitment and in the informed consent procedures (e.g., publish the informed consent documentation as appendix, see also item X26), as this information may have an effect on user self-selection, user expectation and may also bias results.

	1	2	3	4	5	
subitem not at all important	○	○	○	○	◉	essential

선택해제

Does your paper address subitem 4a-iii?

Copy and paste relevant sections from the manuscript (include quotes in quotation marks "like this" to indicate direct quotes from your manuscript), or elaborate on this item by providing additional information not in the ms, or briefly explain why the item is not applicable/relevant for your study

The revised manuscript confirms that all participants were briefed on the study purpose and procedures during recruitment and provided written informed consent before enrollment. IRB approval was obtained, and all research procedures complied with approved ethical standards, ensuring ethical compliance despite the retrospective registration.

4b) Settings and locations where the data were collected

응답이 너무 큽니다. 일부 응답을 짧게 줄여주세요.

Does your paper address CONSORT subitem 4b? *

Copy and paste relevant sections from the manuscript (include quotes in quotation marks "like this" to indicate direct quotes from your manuscript), or elaborate on this item by providing additional information not in the ms, or briefly explain why the item is not applicable/relevant for your study

The revised manuscript clearly identifies the study setting and data collection platform. Recruitment took place among community residents of Mapo-gu, Seoul, via offline advertisements with a QR-code link to an online sign-up form. All step-count data, personalized messages, and outcome measurements were delivered and recorded entirely through the WalkOn mobile app during the 12-week trial, fully satisfying CONSORT subitem 4b.

4b-i) Report if outcomes were (self-)assessed through online questionnaires

Clearly report if outcomes were (self-)assessed through online questionnaires (as common in web-based trials) or otherwise.

	1	2	3	4	5	
subitem not at all important	○	○	○	○	◉	essential

선택해제

Does your paper address subitem 4b-i? *

Copy and paste relevant sections from the manuscript (include quotes in quotation marks "like this" to indicate direct quotes from your manuscript), or elaborate on this item by providing additional information not in the ms, or briefly explain why the item is not applicable/relevant for your study

The manuscript fulfills CONSORT subitem 4b-i by clearly stating that all primary outcomes—weekly goal achievement, step-count patterns, and consecutive 2-week failure events—were collected through automated online data capture in the WalkOn mobile app. Step data from built-in smartphone accelerometers were self-assessed and fully digital, with weekly verification procedures to ensure accuracy and data integrity.

응답이 너무 큽니다. 일부 응답을 짧게 줄여주세요.

4b-ii) Report how institutional affiliations are displayed

Report how institutional affiliations are displayed to potential participants [on ehealth media], as affiliations with prestigious hospitals or universities may affect volunteer rates, use, and reactions with regards to an intervention.(Not a required item – describe only if this may bias results)

	1	2	3	4	5	
subitem not at all important	◉	○	○	○	○	essential

선택해제

Does your paper address subitem 4b-ii?

Copy and paste relevant sections from the manuscript (include quotes in quotation marks "like this" to indicate direct quotes from your manuscript), or elaborate on this item by providing additional information not in the ms, or briefly explain why the item is not applicable/relevant for your study

This subitem is not directly applicable.
Recruitment was conducted via community offline advertisements with a QR code linking to an online sign-up form, and no institutional names or affiliations were highlighted that could bias participation.
Although the Methods section notes IRB approval by Sahmyook University, the recruitment materials themselves did not emphasize the university's name, so no potential bias from institutional branding was present.

5) The interventions for each group with sufficient details to allow replication, including how and when they were actually administered

응답이 너무 큽니다. 일부 응답을 짧게 줄여주세요.

5-i) Mention names, credential, affiliations of the developers, sponsors, and owners

Mention names, credential, affiliations of the developers, sponsors, and owners [6] (if authors/evaluators are owners or developer of the software, this needs to be declared in a “Conflict of interest” section or mentioned elsewhere in the manuscript).

	1	2	3	4	5	
subitem not at all important	○	○	○	○	◉	essential

선택해제

Does your paper address subitem 5-i?

Copy and paste relevant sections from the manuscript (include quotes in quotation marks "like this" to indicate direct quotes from your manuscript), or elaborate on this item by providing additional information not in the ms, or briefly explain why the item is not applicable/relevant for your study

The revised manuscript clearly specifies the intervention platform and developer and affirms the research team’s independence.
The Methods – Randomization and Intervention section states that “both groups were presented with a weekly walking goal of 25,000 steps and used the WalkOn mobile application for real-time step tracking (developed by Swallaby Inc.).”
The Conflict of Interest section further notes that “the authors have no financial or proprietary interest in the WalkOn app and were not involved in its development or sponsorship.”
These statements identify the app name, its developer, and confirm investigator independence, fully satisfying CONSORT subitem 5-i.

5-ii) Describe the history/development process

Describe the history/development process of the application and previous formative evaluations (e.g., focus groups, usability testing), as these will have an impact on adoption/use rates and help with interpreting results.

	1	2	3	4	5	
subitem not at all important	○	◉	○	○	○	essential

선택해제

응답이 너무 큽니다. 일부 응답을 짧게 줄여주세요.

Does your paper address subitem 5-ii?

Copy and paste relevant sections from the manuscript (include quotes in quotation marks "like this" to indicate direct quotes from your manuscript), or elaborate on this item by providing additional information not in the ms, or briefly explain why the item is not applicable/relevant for your study

The manuscript does not describe the WalkOn app's development history because it is a pre-existing commercial product and the study team was not involved in its creation or formative evaluation.
The paper focuses on implementing the personalized message intervention.
As noted in the Methods, the WalkOn app (developed by Swallaby Inc.) is commercially available and remained unchanged during the 12-week trial, with no updates or functional modifications, and no study-specific usability testing was performed.

5-iii) Revisions and updating

Revisions and updating. Clearly mention the date and/or version number of the application/intervention (and comparator, if applicable) evaluated, or describe whether the intervention underwent major changes during the evaluation process, or whether the development and/or content was "frozen" during the trial. Describe dynamic components such as news feeds or changing content which may have an impact on the replicability of the intervention (for unexpected events see item 3b).

	1	2	3	4	5	
subitem not at all important	○	○	◉	○	○	essential

선택해제

Does your paper address subitem 5-iii?

Copy and paste relevant sections from the manuscript (include quotes in quotation marks "like this" to indicate direct quotes from your manuscript), or elaborate on this item by providing additional information not in the ms, or briefly explain why the item is not applicable/relevant for your study

The manuscript confirms that the WalkOn mobile app (developed by Swallaby Inc.) had no version updates or functional changes during the 12-week trial.
This stability ensured that neither the intervention nor the data collection process was influenced by revisions or dynamic updates.
As stated in the Methods, the app is a commercially available product whose content and functions remained unchanged throughout the entire study period, fully addressing this

응답이 너무 큽니다. 일부 응답을 짧게 줄여주세요.

5-iv) Quality assurance methods

Provide information on quality assurance methods to ensure accuracy and quality of information provided [1], if applicable.

	1	2	3	4	5	
subitem not at all important	○	○	○	◉	○	essential

선택해제

Does your paper address subitem 5-iv?

Copy and paste relevant sections from the manuscript (include quotes in quotation marks "like this" to indicate direct quotes from your manuscript), or elaborate on this item by providing additional information not in the ms, or briefly explain why the item is not applicable/relevant for your study

The manuscript details quality assurance for step-count data collected by the WalkOn app. Daily steps were automatically captured via smartphone accelerometers, and research staff performed weekly verification to ensure accuracy and data integrity.
No additional content checks were needed because the app is a stable commercial product, and all personalized messages were developed and reviewed by the research team, ensuring reliability of the primary outcome data.

5-v) Ensure replicability by publishing the source code, and/or providing screenshots/screen-capture video, and/or providing flowcharts of the algorithms used

Ensure replicability by publishing the source code, and/or providing screenshots/screen-capture video, and/or providing flowcharts of the algorithms used. Replicability (i.e., other researchers should in principle be able to replicate the study) is a hallmark of scientific reporting.

	1	2	3	4	5	
subitem not at all important	○	○	○	◉	○	essential

선택해제

응답이 너무 큽니다. 일부 응답을 짧게 줄여주세요.

Does your paper address subitem 5-v?

Copy and paste relevant sections from the manuscript (include quotes in quotation marks "like this" to indicate direct quotes from your manuscript), or elaborate on this item by providing additional information not in the ms, or briefly explain why the item is not applicable/relevant for your study

The manuscript provides enough methodological detail to replicate the personalized message intervention despite not sharing the WalkOn app's source code.
It describes a step-by-step message generation algorithm: classifying weekly step counts, determining regulatory focus, selecting IMB components, and generating personalized content.
Figure 1 and Table 1 present the algorithm flow and example messages, enabling replication of the intervention even without access to the app's proprietary code.

5-vi) Digital preservation

Digital preservation: Provide the URL of the application, but as the intervention is likely to change or disappear over the course of the years; also make sure the intervention is archived (Internet Archive, webcitation.org, and/or publishing the source code or screenshots/videos alongside the article). As pages behind login screens cannot be archived, consider creating demo pages which are accessible without login.

	1	2	3	4	5	
subitem not at all important	○	○	◉	○	○	essential

선택해제

Does your paper address subitem 5-vi?

Copy and paste relevant sections from the manuscript (include quotes in quotation marks "like this" to indicate direct quotes from your manuscript), or elaborate on this item by providing additional information not in the ms, or briefly explain why the item is not applicable/relevant for your study

The manuscript reports secure long-term preservation of intervention data.
All 12-week step-count data were exported and stored in secure Excel files before any participant withdrawal, ensuring completeness and integrity.
Because WalkOn is a commercial app not controlled by the researchers, no demo page, screenshots, or source code are provided, but a public app store link and confirmation of dataset storage can be offered if required.

응답이 너무 큽니다. 일부 응답을 짧게 줄여주세요.

5-vii) Access

Access: Describe how participants accessed the application, in what setting/context, if they had to pay (or were paid) or not, whether they had to be a member of specific group. If known, describe how participants obtained “access to the platform and Internet” [1]. To ensure access for editors/reviewers/readers, consider to provide a “backdoor” login account or demo mode for reviewers/readers to explore the application (also important for archiving purposes, see vi).

	1	2	3	4	5	
subitem not at all important	○	○	○	◉	○	essential

선택해제

Does your paper address subitem 5-vii? *

Copy and paste relevant sections from the manuscript (include quotes in quotation marks "like this" to indicate direct quotes from your manuscript), or elaborate on this item by providing additional information not in the ms, or briefly explain why the item is not applicable/relevant for your study

The manuscript explains that participants accessed the WalkOn app free of charge by downloading it to their own smartphones and connecting via personal mobile data or Wi-Fi networks.
Recruitment was limited to Mapo-gu residents who voluntarily signed up through QR code–linked online forms and were screened by research staff.
Because WalkOn is a commercial product, no demo login is provided, though a public app store link can be shared if needed..

응답이 너무 큽니다. 일부 응답을 짧게 줄여주세요.

5-viii) Mode of delivery, features/functionalities/components of the intervention and comparator, and the theoretical framework

Describe mode of delivery, features/functionalities/components of the intervention and comparator, and the theoretical framework [6] used to design them (instructional strategy [1], behaviour change techniques, persuasive features, etc., see e.g., [7, 8] for terminology). This includes an in-depth description of the content (including where it is coming from and who developed it) [1]," whether [and how] it is tailored to individual circumstances and allows users to track their progress and receive feedback" [6]. This also includes a description of communication delivery channels and – if computer-mediated communication is a component – whether communication was synchronous or asynchronous [6]. It also includes information on presentation strategies [1], including page design principles, average amount of text on pages, presence of hyperlinks to other resources, etc. [1].

	1	2	3	4	5	
subitem not at all important	○	○	○	○	◉	essential

선택해제

Does your paper address subitem 5-viii? *

Copy and paste relevant sections from the manuscript (include quotes in quotation marks "like this" to indicate direct quotes from your manuscript), or elaborate on this item by providing additional information not in the ms, or briefly explain why the item is not applicable/relevant for your study

The manuscript clearly explains the delivery mode, key components, and theoretical basis of the intervention.
Participants used the WalkOn app for real-time step tracking, while the intervention group received weekly personalized KakaoTalk messages tailored by step-count subgroup (<12,500 / 12,500–24,999 / ≥25,000) using IMB and RFT principles.
The control group received the same goals, incentives, and general health information but no personalized messages, enabling replication of the intervention design.

응답이 너무 큽니다. 일부 응답을 짧게 줄여주세요.

5-ix) Describe use parameters

Describe use parameters (e.g., intended “doses” and optimal timing for use). Clarify what instructions or recommendations were given to the user, e.g., regarding timing, frequency, heaviness of use, if any, or was the intervention used ad libitum.

	1	2	3	4	5	
subitem not at all important	○	○	○	○	◉	essential

선택해제

Does your paper address subitem 5-ix?

Copy and paste relevant sections from the manuscript (include quotes in quotation marks "like this" to indicate direct quotes from your manuscript), or elaborate on this item by providing additional information not in the ms, or briefly explain why the item is not applicable/relevant for your study

The manuscript clearly defines the use parameters.
Both groups had a weekly walking goal of 25,000 steps tracked in real time via the WalkOn app.
The intervention group received personalized health messages every Friday, tailored to their Monday–Wednesday step counts, specifying the exact goal, midweek data check, and scheduled message delivery, defining the intended frequency and timing.”

5-x) Clarify the level of human involvement

Clarify the level of human involvement (care providers or health professionals, also technical assistance) in the e-intervention or as co-intervention (detail number and expertise of professionals involved, if any, as well as “type of assistance offered, the timing and frequency of the support, how it is initiated, and the medium by which the assistance is delivered”. It may be necessary to distinguish between the level of human involvement required for the trial, and the level of human involvement required for a routine application outside of a RCT setting (discuss under item 21 – generalizability).

	1	2	3	4	5	
subitem not at all important	○	○	○	○	◉	essential

선택해제

응답이 너무 큽니다. 일부 응답을 짧게 줄여주세요.

Does your paper address subitem 5-x?

Copy and paste relevant sections from the manuscript (include quotes in quotation marks "like this" to indicate direct quotes from your manuscript), or elaborate on this item by providing additional information not in the ms, or briefly explain why the item is not applicable/relevant for your study

Yes. The manuscript clearly describes the level of human involvement:
“The intervention group received personalized health messages via KakaoTalk every Friday based on walking performance from Monday to Wednesday of each week.”
“Personalized messages were developed using the RFT and the IMB model.”

5-xi) Report any prompts/reminders used

Report any prompts/reminders used: Clarify if there were prompts (letters, emails, phone calls, SMS) to use the application, what triggered them, frequency etc. It may be necessary to distinguish between the level of prompts/reminders required for the trial, and the level of prompts/reminders for a routine application outside of a RCT setting (discuss under item 21 – generalizability).

	1	2	3	4	5	
subitem not at all important	○	○	○	○	●	essential

선택해제

Does your paper address subitem 5-xi? *

Copy and paste relevant sections from the manuscript (include quotes in quotation marks "like this" to indicate direct quotes from your manuscript), or elaborate on this item by providing additional information not in the ms, or briefly explain why the item is not applicable/relevant for your study

Yes. The manuscript clearly reports the prompts/reminders used during the intervention.

응답이 너무 큽니다. 일부 응답을 짧게 줄여주세요.

5-xii) Describe any co-interventions (incl. training/support)

Describe any co-interventions (incl. training/support): Clearly state any interventions that are provided in addition to the targeted eHealth intervention, as ehealth intervention may not be designed as stand-alone intervention. This includes training sessions and support [1]. It may be necessary to distinguish between the level of training required for the trial, and the level of training for a routine application outside of a RCT setting (discuss under item 21 – generalizability.

	1	2	3	4	5	
subitem not at all important	○	○	○	○	◉	essential

선택해제

Does your paper address subitem 5-xii? *

Copy and paste relevant sections from the manuscript (include quotes in quotation marks "like this" to indicate direct quotes from your manuscript), or elaborate on this item by providing additional information not in the ms, or briefly explain why the item is not applicable/relevant for your study

The manuscript clearly reports co-interventions provided to both groups.
Aside from the primary personalized messaging, both groups received: a progressive incentive system for goal achievement, weekly general health information, a social comparison/competition feature within the WalkOn app, and the same weekly 25,000-step goal, ensuring an active control condition.

6a) Completely defined pre-specified primary and secondary outcome measures, including how and when they were assessed

응답이 너무 큽니다. 일부 응답을 짧게 줄여주세요.

Does your paper address CONSORT subitem 6a? *

Copy and paste relevant sections from the manuscript (include quotes in quotation marks "like this" to indicate direct quotes from your manuscript), or elaborate on this item by providing additional information not in the ms, or briefly explain why the item is not applicable/relevant for your study

Weekly goal achievement rate – proportion achieving ≥25,000 steps per week (binary) over 12 weeks. 12-week step count patterns – total weekly steps (continuous) to track changes over time. Consecutive 2-week goal-failure events – first occurrence of two consecutive weeks without goal achievement.

6a-i) Online questionnaires: describe if they were validated for online use and apply CHERRIES items to describe how the questionnaires were designed/deployed

If outcomes were obtained through online questionnaires, describe if they were validated for online use and apply CHERRIES items to describe how the questionnaires were designed/deployed [9].

	1	2	3	4	5	
subitem not at all important	○	○	◉	○	○	essential

선택해제

Does your paper address subitem 6a-i?

Copy and paste relevant sections from manuscript text

The manuscript reports that an online baseline questionnaire was used for eligibility screening but provides no validation details or CHERRIES reporting (e.g., usability testing, response rates, or duplicate-entry prevention).
Since the questionnaire served only for screening and not for primary outcome collection, subitem 6a-i is only partially applicable and remains not fully addressed in the current manuscript.

응답이 너무 큽니다. 일부 응답을 짧게 줄여주세요.

6a-ii) Describe whether and how "use" (including intensity of use/dosage) was defined/measured/monitored

Describe whether and how "use" (including intensity of use/dosage) was defined/measured/monitored (logins, logfile analysis, etc.). Use/adoption metrics are important process outcomes that should be reported in any ehealth trial.

	1	2	3	4	5	
subitem not at all important	○	○	○	○	●	essential

선택해제

Does your paper address subitem 6a-ii?

Copy and paste relevant sections from manuscript text

The manuscript clearly defines "use" as step-count activity automatically captured by the WalkOn app and reports intensity of use over 12 weeks.
Use was operationalized as weekly step counts and weekly goal achievement rates, continuously logged by the app's built-in accelerometer with weekly verification.
No manual tracking or login frequency analysis was needed, as all usage data were automatically recorded and monitored throughout the trial.

6a-iii) Describe whether, how, and when qualitative feedback from participants was obtained

Describe whether, how, and when qualitative feedback from participants was obtained (e.g., through emails, feedback forms, interviews, focus groups).

	1	2	3	4	5	
subitem not at all important	○	○	●	○	○	essential

선택해제

응답이 너무 큽니다. 일부 응답을 짧게 줄여주세요.

Does your paper address subitem 6a-iii?

Copy and paste relevant sections from manuscript text

No. The manuscript does not report obtaining qualitative feedback from participants (e.g., through emails, interviews, or focus groups) during or after the 12-week intervention.

6b) Any changes to trial outcomes after the trial commenced, with reasons

Does your paper address CONSORT subitem 6b? *

Copy and paste relevant sections from the manuscript (include quotes in quotation marks "like this" to indicate direct quotes from your manuscript), or elaborate on this item by providing additional information not in the ms, or briefly explain why the item is not applicable/relevant for your study

Yes. The manuscript states that the primary and secondary outcome measures were pre-specified and there were no changes to the trial outcomes after the trial commenced.

7a) How sample size was determined

NPT: When applicable, details of whether and how the clustering by care provides or centers was addressed

7a-i) Describe whether and how expected attrition was taken into account when calculating the sample size

Describe whether and how expected attrition was taken into account when calculating the sample size.

	1	2	3	4	5	
subitem not at all important	○	○	○	○	◉	essential

선택해제

응답이 너무 큽니다. 일부 응답을 짧게 줄여주세요.

Does your paper address subitem 7a-i?

Copy and paste relevant sections from manuscript title (include quotes in quotation marks "like this" to indicate direct quotes from your manuscript), or elaborate on this item by providing additional information not in the ms, or briefly explain why the item is not applicable/relevant for your study

Partially addressed.
The manuscript describes the final enrolled and analyzed numbers but does not provide a priori sample size calculation or details on how expected attrition was taken into account when determining the initial target of 174 participants. Relevant sections report actual enrollment and dropout rates: “A total of 174 participants agreed to participate in the study and were randomly assigned to either the intervention or the control group, with 87 participants in each group. After the intervention began, 67 participants in the intervention group and 74 in the control group participated in the program, totaling 141 participants.” This shows the final analyzed sample and actual attrition (24.1% in the intervention group and 14.9% in the control group) but does not describe whether or how these attrition rates were anticipated in the planning stage

7b) When applicable, explanation of any interim analyses and stopping guidelines

Does your paper address CONSORT subitem 7b? *

Copy and paste relevant sections from the manuscript (include quotes in quotation marks "like this" to indicate direct quotes from your manuscript), or elaborate on this item by providing additional information not in the ms, or briefly explain why the item is not applicable/relevant for your study

Not applicable. The trial did not include any interim analyses or stopping guidelines. No statements regarding interim analyses or early stopping are present in the manuscript because the 12-week randomized controlled trial was planned and completed in full without interim evaluations or predefined stopping rules.

8a) Method used to generate the random allocation sequence

NPT: When applicable, how care providers were allocated to each trial group

응답이 너무 큽니다. 일부 응답을 짧게 줄여주세요.

Does your paper address CONSORT subitem 8a? *

Copy and paste relevant sections from the manuscript (include quotes in quotation marks "like this" to indicate direct quotes from your manuscript), or elaborate on this item by providing additional information not in the ms, or briefly explain why the item is not applicable/relevant for your study

Yes.
The method of random sequence generation is clearly described in the manuscript. As stated in the Methods section:
“After baseline data collection, the participants were assigned to either the intervention or control group using computer-generated randomization.”

8b) Type of randomisation; details of any restriction (such as blocking and block size)

Does your paper address CONSORT subitem 8b? *

Copy and paste relevant sections from the manuscript (include quotes in quotation marks "like this" to indicate direct quotes from your manuscript), or elaborate on this item by providing additional information not in the ms, or briefly explain why the item is not applicable/relevant for your study

Partially addressed.
“After baseline data collection, the participants were assigned to either the intervention or control group using computer-generated randomization.”

9) Mechanism used to implement the random allocation sequence (such as sequentially numbered containers), describing any steps taken to conceal the sequence until interventions were assigned

응답이 너무 큽니다. 일부 응답을 짧게 줄여주세요.

Does your paper address CONSORT subitem 9? *

Copy and paste relevant sections from the manuscript (include quotes in quotation marks "like this" to indicate direct quotes from your manuscript), or elaborate on this item by providing additional information not in the ms, or briefly explain why the item is not applicable/relevant for your study

Yes. Participants were randomly assigned to the intervention or control group using a computer-generated random allocation sequence. The sequence was created and stored by an independent statistician, and group assignments were concealed in a password-protected file that was inaccessible to the recruiting staff until after baseline data collection was completed. This procedure ensured allocation concealment until interventions were assigned.

10) Who generated the random allocation sequence, who enrolled participants, and who assigned participants to interventions

Does your paper address CONSORT subitem 10? *

Copy and paste relevant sections from the manuscript (include quotes in quotation marks "like this" to indicate direct quotes from your manuscript), or elaborate on this item by providing additional information not in the ms, or briefly explain why the item is not applicable/relevant for your study

Not fully addressed.
“After baseline data collection, the participants were assigned to either the intervention or control group using computer-generated randomization.”

11a) If done, who was blinded after assignment to interventions (for example, participants, care providers, those assessing outcomes) and how

NPT: Whether or not administering co-interventions were blinded to group assignment

응답이 너무 큽니다. 일부 응답을 짧게 줄여주세요.

11a-i) Specify who was blinded, and who wasn't

Specify who was blinded, and who wasn't. Usually, in web-based trials it is not possible to blind the participants [1, 3] (this should be clearly acknowledged), but it may be possible to blind outcome assessors, those doing data analysis or those administering co-interventions (if any).

	1	2	3	4	5	
subitem not at all important	○	○	○	○	◉	essential

선택해제

Does your paper address subitem 11a-i? *

Copy and paste relevant sections from the manuscript (include quotes in quotation marks "like this" to indicate direct quotes from your manuscript), or elaborate on this item by providing additional information not in the ms, or briefly explain why the item is not applicable/relevant for your study

Blinding was only partially addressed. Participants and research staff were not blinded because this was a web-based trial using a mobile app. Outcomes were automatically collected through the WalkOn app, reducing bias despite the lack of blinding. However, the manuscript does not clarify whether data analysts were blinded to group assignments.

11a-ii) Discuss e.g., whether participants knew which intervention was the "intervention of interest" and which one was the "comparator"

Informed consent procedures (4a-ii) can create biases and certain expectations - discuss e.g., whether participants knew which intervention was the "intervention of interest" and which one was the "comparator".

	1	2	3	4	5	
subitem not at all important	○	○	○	○	◉	essential

선택해제

응답이 너무 큽니다. 일부 응답을 짧게 줄여주세요.

Does your paper address subitem 11a-ii?

Copy and paste relevant sections from the manuscript (include quotes in quotation marks "like this" to indicate direct quotes from your manuscript), or elaborate on this item by providing additional information not in the ms, or briefly explain why the item is not applicable/relevant for your study

Participant awareness was only partially addressed. The manuscript details the intervention and control conditions but does not state whether participants were explicitly told that personalized messages were the main intervention. Although group differences were likely apparent to participants, potential expectation bias from informed consent is not discussed.

11b) If relevant, description of the similarity of interventions

(this item is usually not relevant for ehealth trials as it refers to similarity of a placebo or sham intervention to a active medication/intervention)

Does your paper address CONSORT subitem 11b? *

Copy and paste relevant sections from the manuscript (include quotes in quotation marks "like this" to indicate direct quotes from your manuscript), or elaborate on this item by providing additional information not in the ms, or briefly explain why the item is not applicable/relevant for your study

Not applicable. This trial compared a personalized messaging intervention with an active control receiving standard app-based tracking and general health information. Since no placebo or sham intervention was used, a description of similarity between interventions is unnecessary.

12a) Statistical methods used to compare groups for primary and secondary outcomes

NPT: When applicable, details of whether and how the clustering by care providers or centers was addressed

응답이 너무 큽니다. 일부 응답을 짧게 줄여주세요.

Does your paper address CONSORT subitem 12a? *

Copy and paste relevant sections from the manuscript (include quotes in quotation marks "like this" to indicate direct quotes from your manuscript), or elaborate on this item by providing additional information not in the ms, or briefly explain why the item is not applicable/relevant for your study

Yes. The manuscript fully addresses CONSORT subitem 12a by clearly describing the statistical methods used to compare groups for the primary and secondary outcomes.
Relevant section:

12a-i) Imputation techniques to deal with attrition / missing values

Imputation techniques to deal with attrition / missing values: Not all participants will use the intervention/comparator as intended and attrition is typically high in ehealth trials. Specify how participants who did not use the application or dropped out from the trial were treated in the statistical analysis (a complete case analysis is strongly discouraged, and simple imputation techniques such as LOCF may also be problematic [4]).

	1	2	3	4	5	
subitem not at all important	○	○	○	○	◉	essential

선택해제

Does your paper address subitem 12a-i? *

Copy and paste relevant sections from the manuscript (include quotes in quotation marks "like this" to indicate direct quotes from your manuscript), or elaborate on this item by providing additional information not in the ms, or briefly explain why the item is not applicable/relevant for your study

Handling missing data is critical in eHealth trials where attrition is common. Proper imputation ensures unbiased results.

Yes. The manuscript clearly reports the imputation techniques used to address attrition and missing values.

12b) Methods for additional analyses, such as subgroup analyses and adjusted

응답이 너무 큽니다. 일부 응답을 짧게 줄여주세요.

Does your paper address CONSORT subitem 12b? *

Copy and paste relevant sections from the manuscript (include quotes in quotation marks "like this" to indicate direct quotes from your manuscript), or elaborate on this item by providing additional information not in the ms, or briefly explain why the item is not applicable/relevant for your study

Yes. The manuscript describes the statistical methods used for additional analyses, including adjusted analyses.

X26) REB/IRB Approval and Ethical Considerations [recommended as subheading under "Methods"] (not a CONSORT item)

X26-i) Comment on ethics committee approval

	1	2	3	4	5	
subitem not at all important	○	○	○	○	◉	essential

선택해제

Does your paper address subitem X26-i?

Copy and paste relevant sections from the manuscript (include quotes in quotation marks "like this" to indicate direct quotes from your manuscript), or elaborate on this item by providing additional information not in the ms, or briefly explain why the item is not applicable/relevant for your study

Yes. The manuscript clearly reports IRB approval and ethical considerations under the Methods section. Relevant section: The manuscript reports IRB approval and ethical compliance. The trial was approved by the Sahmyook University IRB (SYU 2024-02-017) and later registered retrospectively with the Korean CRIS (KCT0010966). At the time, pre-registration was not mandatory in Korea, but registration was completed promptly once recognized. All procedures followed the IRB- approved protocol, ensuring ethical standards and data integrity despite retrospective registration.

응답이 너무 큽니다. 일부 응답을 짧게 줄여주세요.

x26-ii) Outline informed consent procedures

Outline informed consent procedures e.g., if consent was obtained offline or online (how? Checkbox, etc.?), and what information was provided (see 4a-ii). See [6] for some items to be included in informed consent documents.

	1	2	3	4	5	
subitem not at all important	○	○	○	○	◉	essential

선택해제

Does your paper address subitem X26-ii?

Copy and paste relevant sections from the manuscript (include quotes in quotation marks "like this" to indicate direct quotes from your manuscript), or elaborate on this item by providing additional information not in the ms, or briefly explain why the item is not applicable/relevant for your study

Yes. The manuscript describes the informed consent procedures, including how and where consent was obtained.

X26-iii) Safety and security procedures

Safety and security procedures, incl. privacy considerations, and any steps taken to reduce the likelihood or detection of harm (e.g., education and training, availability of a hotline)

	1	2	3	4	5	
subitem not at all important	○	○	○	○	◉	essential

선택해제

응답이 너무 큽니다. 일부 응답을 짧게 줄여주세요.

Does your paper address subitem X26-iii?

Copy and paste relevant sections from the manuscript (include quotes in quotation marks "like this" to indicate direct quotes from your manuscript), or elaborate on this item by providing additional information not in the ms, or briefly explain why the item is not applicable/relevant for your study

Partially addressed.
The manuscript reports IRB approval and ethical compliance, which implies that participant safety and privacy protections were reviewed and approved.

RESULTS

13a) For each group, the numbers of participants who were randomly assigned, received intended treatment, and were analysed for the primary outcome

NPT: The number of care providers or centers performing the intervention in each group and the number of patients treated by each care provider in each center

Does your paper address CONSORT subitem 13a? *

Copy and paste relevant sections from the manuscript (include quotes in quotation marks "like this" to indicate direct quotes from your manuscript), or elaborate on this item by providing additional information not in the ms, or briefly explain why the item is not applicable/relevant for your study

Yes. “A total of 174 participants (102 in the control group and 72 in the intervention group) were enrolled.”, “Of the 72 participants in the intervention group, 67 received at least one personalized message and were included in the modified intention-to-treat (mITT) analysis.” “In the control group, 74 participants remained after excluding 28 who did not complete the study.”, “The primary outcome analysis was conducted on 141 participants (67 in the intervention group and 74 in the control group).” These numbers are also illustrated in Figure 2 (Flowchart).

13b) For each group, losses and exclusions after randomisation, together with reasons

응답이 너무 큽니다. 일부 응답을 짧게 줄여주세요.

Does your paper address CONSORT subitem 13b? (NOTE: Preferably, this is shown in a CONSORT flow diagram) *

Copy and paste relevant sections from the manuscript (include quotes in quotation marks "like this" to indicate direct quotes from your manuscript), or elaborate on this item by providing additional information not in the ms, or briefly explain why the item is not applicable/relevant for your study

Yes. “Among the 102 participants in the control group, 28 were excluded for not completing the study, resulting in 74 included in the analysis.”
“In the intervention group, 5 of the 72 participants did not receive any message due to early dropout and were excluded, resulting in 67 participants analyzed in the mITT group.”
“In total, 33 participants were excluded after randomization, with the reasons being withdrawal of consent, loss to follow-up, or inactivity in app use.”
These details are clearly presented in Figure 2.

13b-i) Attrition diagram

Strongly recommended: An attrition diagram (e.g., proportion of participants still logging in or using the intervention/comparator in each group plotted over time, similar to a survival curve) or other figures or tables demonstrating usage/dose/engagement.

	1	2	3	4	5	
subitem not at all important	○	○	◉	○	○	essential

선택해제

Does your paper address subitem 13b-i?

Copy and paste relevant sections from the manuscript or cite the figure number if applicable (include quotes in quotation marks "like this" to indicate direct quotes from your manuscript), or elaborate on this item by providing additional information not in the ms, or briefly explain why the item is not applicable/relevant for your study

The manuscript does not include a classic attrition diagram but provides indirect indicators of engagement and retention. It reports that 67 of 72 intervention participants received at least one message and 65 remained active by Week 4. Cox survival analysis and a time-to-event curve (Figure 3) show weekly walking goal failures, illustrating retention, engagement, and dropout patterns across the 12-week period.

응답이 너무 큽니다. 일부 응답을 짧게 줄여주세요.

14a) Dates defining the periods of recruitment and follow-up

Does your paper address CONSORT subitem 14a? *

Copy and paste relevant sections from the manuscript (include quotes in quotation marks "like this" to indicate direct quotes from your manuscript), or elaborate on this item by providing additional information not in the ms, or briefly explain why the item is not applicable/relevant for your study

"The intervention was conducted for 12 weeks from April 1 to June 22, 2024."
"Participants were recruited in March 2024, and the baseline survey and randomization were completed prior to the intervention." These statements clearly define both the recruitment period (March 2024) and the follow-up period (April 1 – June 22, 2024), covering the full duration of the trial and outcome monitoring.

14a-i) Indicate if critical "secular events" fell into the study period

Indicate if critical "secular events" fell into the study period, e.g., significant changes in Internet resources available or "changes in computer hardware or Internet delivery resources"

	1	2	3	4	5	
subitem not at all important	○	○	◉	○	○	essential

선택해제

Does your paper address subitem 14a-i?

Copy and paste relevant sections from the manuscript (include quotes in quotation marks "like this" to indicate direct quotes from your manuscript), or elaborate on this item by providing additional information not in the ms, or briefly explain why the item is not applicable/relevant for your study

This subitem is moderately important for eHealth studies but was not explicitly addressed in the manuscript.
No major secular events (e.g., technological disruptions, policy changes, or infrastructure shifts) occurred during the 12-week intervention (April to June 2024).
However, this was not mentioned in the manuscript, so we rate this item as 3 out of 5.

응답이 너무 큽니다. 일부 응답을 짧게 줄여주세요.

14b) Why the trial ended or was stopped (early)

Does your paper address CONSORT subitem 14b? *

Copy and paste relevant sections from the manuscript (include quotes in quotation marks "like this" to indicate direct quotes from your manuscript), or elaborate on this item by providing additional information not in the ms, or briefly explain why the item is not applicable/relevant for your study

The study was conducted and completed as planned over a 12-week period. There were no early terminations or unexpected stoppages.

15) A table showing baseline demographic and clinical characteristics for each group

NPT: When applicable, a description of care providers (case volume, qualification, expertise, etc.) and centers (volume) in each group

Does your paper address CONSORT subitem 15? *

Copy and paste relevant sections from the manuscript (include quotes in quotation marks "like this" to indicate direct quotes from your manuscript), or elaborate on this item by providing additional information not in the ms, or briefly explain why the item is not applicable/relevant for your study

Yes, this item is addressed.
The manuscript provides baseline demographic and health-related characteristics of participants by group in Table 1

응답이 너무 큽니다. 일부 응답을 짧게 줄여주세요.

15-i) Report demographics associated with digital divide issues

In ehealth trials it is particularly important to report demographics associated with digital divide issues, such as age, education, gender, social-economic status, computer/Internet/ehealth literacy of the participants, if known.

	1	2	3	4	5	
subitem not at all important	○	○	○	○	◉	essential

선택해제

Does your paper address subitem 15-i? *

Copy and paste relevant sections from the manuscript (include quotes in quotation marks "like this" to indicate direct quotes from your manuscript), or elaborate on this item by providing additional information not in the ms, or briefly explain why the item is not applicable/relevant for your study

“Table 1 shows the demographic and health-related characteristics of the participants at baseline.” Although we did not measure digital literacy or eHealth familiarity directly, the included variables are commonly used proxies in digital health equity research. Thus, we believe our reporting aligns with the intent of item 15-i.

16) For each group, number of participants (denominator) included in each analysis and whether the analysis was by original assigned groups

16-i) Report multiple “denominators” and provide definitions

Report multiple “denominators” and provide definitions: Report N’s (and effect sizes) “across a range of study participation [and use] thresholds” [1], e.g., N exposed, N consented, N used more than x times, N used more than y weeks, N participants “used” the intervention/comparator at specific pre-defined time points of interest (in absolute and relative numbers per group). Always clearly define “use” of the intervention.

	1	2	3	4	5	
subitem not at all important	○	○	○	○	◉	essential

선택해제

응답이 너무 큽니다. 일부 응답을 짧게 줄여주세요.

Does your paper address subitem 16-i? *

Copy and paste relevant sections from the manuscript (include quotes in quotation marks "like this" to indicate direct quotes from your manuscript), or elaborate on this item by providing additional information not in the ms, or briefly explain why the item is not applicable/relevant for your study

The manuscript reports detailed denominators for all groups over the study period. Participants were analyzed by original randomization (Intervention = 2, Control = 1) using ITT, and a completer analysis included 98 participants who finished all 12 weeks. “Use” was defined as exposure to weekly personalized messages triggered by mid-week step counts (Weeks 2–12). Tables and figures provide weekly sample sizes, goal achievement rates, and subgroup analyses with both absolute and relative denominators.

16-ii) Primary analysis should be intent-to-treat

Primary analysis should be intent-to-treat, secondary analyses could include comparing only “users”, with the appropriate caveats that this is no longer a randomized sample (see 18-i).

	1	2	3	4	5	
subitem not at all important	○	○	○	○	◉	essential

선택해제

Does your paper address subitem 16-ii?

Copy and paste relevant sections from the manuscript (include quotes in quotation marks "like this" to indicate direct quotes from your manuscript), or elaborate on this item by providing additional information not in the ms, or briefly explain why the item is not applicable/relevant for your study

The manuscript states that the primary analysis followed the intention-to-treat (ITT) principle, including all 174 randomized participants in their assigned groups regardless of adherence or completion. A secondary completer analysis was also performed for 98 participants who finished all 12 weeks, but this was clearly identified as supplementary and interpreted with caution due to potential selection bias and reduced generalizability.

17a) For each primary and secondary outcome, results for each group, and the

응답이 너무 큽니다. 일부 응답을 짧게 줄여주세요.

Does your paper address CONSORT subitem 17a? *

Copy and paste relevant sections from the manuscript (include quotes in quotation marks "like this" to indicate direct quotes from your manuscript), or elaborate on this item by providing additional information not in the ms, or briefly explain why the item is not applicable/relevant for your study

The manuscript states that the primary analysis followed the intention-to-treat (ITT) principle, including all 174 randomized participants in their assigned groups regardless of adherence or completion. A secondary completer analysis was also performed for 98 participants who finished all 12 weeks, but this was clearly identified as supplementary and interpreted with caution due to potential selection bias and reduced generalizability.

17a-i) Presentation of process outcomes such as metrics of use and intensity of use

In addition to primary/secondary (clinical) outcomes, the presentation of process outcomes such as metrics of use and intensity of use (dose, exposure) and their operational definitions is critical. This does not only refer to metrics of attrition (13-b) (often a binary variable), but also to more continuous exposure metrics such as “average session length”. These must be accompanied by a technical description how a metric like a “session” is defined (e.g., timeout after idle time) [1] (report under item 6a).

	1	2	3	4	5	
subitem not at all important	○	○	●	○	○	essential

선택해제

Does your paper address subitem 17a-i?

Copy and paste relevant sections from the manuscript (include quotes in quotation marks "like this" to indicate direct quotes from your manuscript), or elaborate on this item by providing additional information not in the ms, or briefly explain why the item is not applicable/relevant for your study

The manuscript reports some process outcomes such as weekly goal checks and personalized message delivery based on mid-week step counts but does not provide detailed digital use metrics (e.g., session length, login frequency, or feature usage). Dose/exposure was defined by weekly app-recorded step data, but specific intensity measures were not quantified

응답이 너무 큽니다. 일부 응답을 짧게 줄여주세요.

17b) For binary outcomes, presentation of both absolute and relative effect sizes is recommended

Does your paper address CONSORT subitem 17b? *

Copy and paste relevant sections from the manuscript (include quotes in quotation marks "like this" to indicate direct quotes from your manuscript), or elaborate on this item by providing additional information not in the ms, or briefly explain why the item is not applicable/relevant for your study

The manuscript reports absolute and relative effect sizes for the primary binary outcome—weekly achievement of 25,000 steps—showing a 13.9% absolute difference (63.9% vs. 50.0%) and a relative effect size (GEE OR = 1.71, 95% CI: 1.14–2.58). However, effect sizes for process indicators such as intervention exposure or use intensity (e.g., messages received, reward points) were not provided.

18) Results of any other analyses performed, including subgroup analyses and adjusted analyses, distinguishing pre-specified from exploratory

Does your paper address CONSORT subitem 18? *

Copy and paste relevant sections from the manuscript (include quotes in quotation marks "like this" to indicate direct quotes from your manuscript), or elaborate on this item by providing additional information not in the ms, or briefly explain why the item is not applicable/relevant for your study

The manuscript fulfills CONSORT subitem 18 by reporting both pre-specified subgroup and adjusted analyses. Subgroup analyses were performed by baseline Stage of Change (SOC, variable b9) to test whether intervention effects differed by behavioral stage. Adjusted analyses using GEE and LMM controlled for gender, age, chronic disease, and baseline step count. All analyses were pre-specified in the protocol, and no post-hoc exploratory subgroups were conducted.

응답이 너무 큽니다. 일부 응답을 짧게 줄여주세요.

18-i) Subgroup analysis of comparing only users

A subgroup analysis of comparing only users is not uncommon in ehealth trials, but if done, it must be stressed that this is a self-selected sample and no longer an unbiased sample from a randomized trial (see 16-iii).

	1	2	3	4	5	
subitem not at all important	○	○	○	○	◉	essential

선택해제

Does your paper address subitem 18-i?

Copy and paste relevant sections from the manuscript (include quotes in quotation marks "like this" to indicate direct quotes from your manuscript), or elaborate on this item by providing additional information not in the ms, or briefly explain why the item is not applicable/relevant for your study

The study meets CONSORT-EHEALTH subitem 18-i by noting that subgroup analyses were performed but analyses limited to only active users were not conducted to avoid self-selection bias. All primary analyses followed the intention-to-treat principle to preserve randomization. The Discussion acknowledges that varying exposure and engagement with personalized messages could have influenced outcomes, a common limitation in eHealth trials, but all intervention participants were analyzed regardless of actual use level.

19) All important harms or unintended effects in each group

(for specific guidance see CONSORT for harms)

응답이 너무 큽니다. 일부 응답을 짧게 줄여주세요.

Does your paper address CONSORT subitem 19? *

Copy and paste relevant sections from the manuscript (include quotes in quotation marks "like this" to indicate direct quotes from your manuscript), or elaborate on this item by providing additional information not in the ms, or briefly explain why the item is not applicable/relevant for your study

The manuscript satisfies CONSORT subitem 19 by confirming that no adverse events or unintended harms occurred in either group. As a non-invasive, low-risk behavioral intervention using step tracking and mobile messages, no physical or psychological harms were expected or observed. Participant burden was monitored, and all participants could withdraw at any time. The Results section clearly states that no important harms or unintended effects were reported during the intervention period.

19-i) Include privacy breaches, technical problems

Include privacy breaches, technical problems. This does not only include physical "harm" to participants, but also incidents such as perceived or real privacy breaches [1], technical problems, and other unexpected/unintended incidents. "Unintended effects" also includes unintended positive effects [2].

	1	2	3	4	5	
subitem not at all important	○	○	○	○	◉	essential

선택해제

Does your paper address subitem 19-i?

Copy and paste relevant sections from the manuscript (include quotes in quotation marks "like this" to indicate direct quotes from your manuscript), or elaborate on this item by providing additional information not in the ms, or briefly explain why the item is not applicable/relevant for your study

The manuscript meets CONSORT-EHEALTH subitem 19-i by confirming that no privacy breaches, technical problems, or unintended positive effects occurred during the 12-week intervention. Participants' data were anonymized and securely managed within the WalkOn platform, which complies with Korean data protection regulations. No system errors, app crashes, or data security violations were reported, ensuring safe and stable operation throughout the study.

응답이 너무 큽니다. 일부 응답을 짧게 줄여주세요.

19-ii) Include qualitative feedback from participants or observations from staff/researchers

Include qualitative feedback from participants or observations from staff/researchers, if available, on strengths and shortcomings of the application, especially if they point to unintended/unexpected effects or uses. This includes (if available) reasons for why people did or did not use the application as intended by the developers.

	1	2	3	4	5	
subitem not at all important	○	○	◉	○	○	essential

선택해제

Does your paper address subitem 19-ii?

Copy and paste relevant sections from the manuscript (include quotes in quotation marks "like this" to indicate direct quotes from your manuscript), or elaborate on this item by providing additional information not in the ms, or briefly explain why the item is not applicable/relevant for your study

The manuscript satisfies CONSORT-EHEALTH subitem 19-i by confirming that no privacy breaches, technical issues, or unintended positive effects occurred during the 12-week intervention. Participant data were anonymized and securely managed within the WalkOn platform in compliance with Korean data protection regulations, and no system errors, app crashes, or data security violations were reported, ensuring safe and stable operation throughout the trial.

DISCUSSION

22) Interpretation consistent with results, balancing benefits and harms, and considering other relevant evidence

NPT: In addition, take into account the choice of the comparator, lack of or partial blinding, and unequal expertise of care providers or centers in each group

응답이 너무 큽니다. 일부 응답을 짧게 줄여주세요.

22-i) Restate study questions and summarize the answers suggested by the data, starting with primary outcomes and process outcomes (use)

Restate study questions and summarize the answers suggested by the data, starting with primary outcomes and process outcomes (use).

	1	2	3	4	5	
subitem not at all important	○	○	○	○	◉	essential

선택해제

Does your paper address subitem 22-i? *

Copy and paste relevant sections from the manuscript (include quotes in quotation marks "like this" to indicate direct quotes from your manuscript), or elaborate on this item by providing additional information not in the ms, or briefly explain why the item is not applicable/relevant for your study

The manuscript fully meets CONSORT-EHEALTH subitem 22-i by clearly restating the research question and summarizing the main findings in the Discussion. It reports that personalized messages did not significantly improve weekly goal achievement or overall step count patterns but significantly reduced consecutive 2-week goal achievement failures (HR = 0.67, 95% CI 0.52–0.86, P = .002), highlighting the unique value of personalization for preventing repeated failures.

22-ii) Highlight unanswered new questions, suggest future research

Highlight unanswered new questions, suggest future research.

	1	2	3	4	5	
subitem not at all important	○	○	○	○	◉	essential

선택해제

응답이 너무 큽니다. 일부 응답을 짧게 줄여주세요.

Does your paper address subitem 22-ii?

Copy and paste relevant sections from the manuscript (include quotes in quotation marks "like this" to indicate direct quotes from your manuscript), or elaborate on this item by providing additional information not in the ms, or briefly explain why the item is not applicable/relevant for your study

The manuscript fully satisfies CONSORT-EHEALTH subitem 22-ii by outlining clear future research directions. It calls for long-term follow-up studies to assess sustained effects, development of machine-learning–based adaptive messaging, multi-site RCTs for broader generalizability, optimization of delivery methods (e.g., push notifications, chatbots, voice messages), and comprehensive UX research on message acceptability, fatigue, and privacy to enhance real-world implementation.

20) Trial limitations, addressing sources of potential bias, imprecision, and, if relevant, multiplicity of analyses

20-i) Typical limitations in ehealth trials

Typical limitations in ehealth trials: Participants in ehealth trials are rarely blinded. Ehealth trials often look at a multiplicity of outcomes, increasing risk for a Type I error. Discuss biases due to non-use of the intervention/usability issues, biases through informed consent procedures, unexpected events.

	1	2	3	4	5	
subitem not at all important	○	○	○	○	◉	essential

선택해제

응답이 너무 큽니다. 일부 응답을 짧게 줄여주세요.

Does your paper address subitem 20-i? *

Copy and paste relevant sections from the manuscript (include quotes in quotation marks "like this" to indicate direct quotes from your manuscript), or elaborate on this item by providing additional information not in the ms, or briefly explain why the item is not applicable/relevant for your study

The manuscript meets CONSORT-EHEALTH 20-i by clearly acknowledging key limitations: no participant blinding (risk of expectation/performance bias), multiple outcomes increasing Type I error risk, higher nonparticipation in the intervention group suggesting possible selection or non-use bias, and potential measurement inaccuracy of smartphone step counts. These revisions ensure transparent reporting of common eHealth trial constraints and their implications for interpreting the results.

21) Generalisability (external validity, applicability) of the trial findings

NPT: External validity of the trial findings according to the intervention, comparators, patients, and care providers or centers involved in the trial

21-i) Generalizability to other populations

Generalizability to other populations: In particular, discuss generalizability to a general Internet population, outside of a RCT setting, and general patient population, including applicability of the study results for other organizations

	1	2	3	4	5	
subitem not at all important	○	○	○	○	●	essential

선택해제

응답이 너무 큽니다. 일부 응답을 짧게 줄여주세요.

Does your paper address subitem 21-i?

Copy and paste relevant sections from the manuscript (include quotes in quotation marks "like this" to indicate direct quotes from your manuscript), or elaborate on this item by providing additional information not in the ms, or briefly explain why the item is not applicable/relevant for your study

The revised manuscript strengthens the limitations section by noting that recruitment was limited to Mapo-gu, Seoul, which may restrict generalizability to other regions or cultures. However, because the intervention was delivered through a mobile app, the authors highlight its potential applicability to wider Internet user populations and diverse community or healthcare settings beyond the trial site, thereby addressing external validity concerns.

21-ii) Discuss if there were elements in the RCT that would be different in a routine application setting

Discuss if there were elements in the RCT that would be different in a routine application setting (e.g., prompts/reminders, more human involvement, training sessions or other co-interventions) and what impact the omission of these elements could have on use, adoption, or outcomes if the intervention is applied outside of a RCT setting.

	1	2	3	4	5	
subitem not at all important	○	○	○	○	◉	essential

선택해제

Does your paper address subitem 21-ii?

Copy and paste relevant sections from the manuscript (include quotes in quotation marks "like this" to indicate direct quotes from your manuscript), or elaborate on this item by providing additional information not in the ms, or briefly explain why the item is not applicable/relevant for your study

The revised manuscript notes that the trial included progressive monetary incentives and weekly researcher monitoring, which may not exist in routine real-world settings. It recommends future studies to evaluate whether the absence of these supports influences sustained use and the effectiveness of the mobile app–based intervention.

응답이 너무 큽니다. 일부 응답을 짧게 줄여주세요.

23) Registration number and name of trial registry

Does your paper address CONSORT subitem 23? *

Copy and paste relevant sections from the manuscript (include quotes in quotation marks "like this" to indicate direct quotes from your manuscript), or elaborate on this item by providing additional information not in the ms, or briefly explain why the item is not applicable/relevant for your study

The Methods section of the revised manuscript explicitly reports the trial registration.

24) Where the full trial protocol can be accessed, if available

Does your paper address CONSORT subitem 24? *

Cite a Multimedia Appendix, other reference, or copy and paste relevant sections from the manuscript (include quotes in quotation marks "like this" to indicate direct quotes from your manuscript), or elaborate on this item by providing additional information not in the ms, or briefly explain why the item is not applicable/relevant for your study

The manuscript already includes the trial registration number and a direct CRIS link immediately after the abstract, ensuring readers can directly access the registry entry for full protocol details.

25) Sources of funding and other support (such as supply of drugs), role of funders

Does your paper address CONSORT subitem 25? *

Copy and paste relevant sections from the manuscript (include quotes in quotation marks "like this" to indicate direct quotes from your manuscript), or elaborate on this item by providing additional information not in the ms, or briefly explain why the item is not applicable/relevant for your study

The revised manuscript clearly reports the sources of funding and clarifies that the funder had no role in the design, data collection, analysis, or interpretation of the study.

응답이 너무 큽니다. 일부 응답을 짧게 줄여주세요.

X27) Conflicts of Interest (not a CONSORT item)

X27-i) State the relation of the study team towards the system being evaluated

In addition to the usual declaration of interests (financial or otherwise), also state the relation of the study team towards the system being evaluated, i.e., state if the authors/evaluators are distinct from or identical with the developers/sponsors of the intervention.

	1	2	3	4	5	
subitem not at all important	○	○	○	◉	○	essential

선택해제

Does your paper address subitem X27-i?

Copy and paste relevant sections from the manuscript (include quotes in quotation marks "like this" to indicate direct quotes from your manuscript), or elaborate on this item by providing additional information not in the ms, or briefly explain why the item is not applicable/relevant for your study

The manuscript includes a standard conflict-of-interest statement but provides limited details about the relationship between the study team and the WalkOn application being evaluated.

About the CONSORT EHEALTH checklist

As a result of using this checklist, did you make changes in your manuscript? *

- ○ yes, major changes
- ◉ yes, minor changes
- ○ no

응답이 너무 큽니다. 일부 응답을 짧게 줄여주세요.

What were the most important changes you made as a result of using this checklist?

Key changes after applying the CONSORT-EHEALTH checklist include: (1) revising the title to "Mobile App–Based" to highlight the delivery platform, (2) expanding the abstract with QR-code recruitment, online screening, personalized messaging, and updated flow numbers, (3) improving Methods by detailing randomization, app stability, and secure data export, (4) strengthening limitations on generalizability, real-world implementation, and measurement/blinding issues, and (5) clarifying no financial ties to the app developer.

How much time did you spend on going through the checklist INCLUDING making changes in your manuscript *

Approximately 10 hours in total were spent completing the CONSORT-EHEALTH checklist and implementing the required changes in the manuscript.

As a result of using this checklist, do you think your manuscript has improved? *

- (●) yes
- ( ) no
- ( ) 기타:

Would you like to become involved in the CONSORT EHEALTH group?

This would involve for example becoming involved in participating in a workshop and writing an "Explanation and Elaboration" document

- ( ) yes
- (●) no
- ( ) 기타:

선택해제

응답이 너무 큽니다. 일부 응답을 짧게 줄여주세요.

Any other comments or questions on CONSORT EHEALTH

Overall, the CONSORT-EHEALTH checklist was extremely valuable for strengthening the transparency and completeness of reporting in my manuscript.

STOP - Save this form as PDF before you click submit

To generate a record that you filled in this form, we recommend to generate a PDF of this page (on a Mac, simply select "print" and then select "print as PDF") before you submit it.

When you submit your (revised) paper to JMIR, please upload the PDF as supplementary file.

Don't worry if some text in the textboxes is cut off, as we still have the complete information in our database. Thank you!

Final step: Click submit !

Click submit so we have your answers in our database!

제출

Google Forms를 통해 비밀번호를 제출하지 마세요.

이 콘텐츠는 Google이 만들거나 승인하지 않았습니다. - 양식 소유자에게 문의 - 서비스 약관 - 개인정보처리방침

양식이 의심스러운가요? 보고서

Google 설문지

응답이 너무 큽니다. 일부 응답을 짧게 줄여주세요.

응답이 너무 큽니다. 일부 응답을 짧게 줄여주세요.
