# Supplementary material for: Personalized Mobile App–Based Messaging and Step Tracking for Improving Walking Behavior in Adults: Randomized Controlled Trial
Source: JMIR Mhealth Uhealth. 2026 Sep 17;14:e84430. doi: 10.2196/84430 (PMC13631492; doi:10.2196/84430)
Supplement: Supplementary file 2 [file mhealth_v14i1e84430_app1.docx]

Multimedia Appendix 2. Examples of Personalized Messages Sent by Performance Level

Low Performance Message Example: "Hello [Name]! Mid-week check results (as of Thursday 0:00): Please keep working toward achieving this week's goal (25,000 steps per week)!! How about checking your step count during lunch time every day? It will be more motivating. ^^ Even though you might be tired on weekends, if you come outside and enjoy the warm spring day, you'll be able to secure 500 points quickly. [Name]! Please keep trying a little more. I'll visit again next week. Thank you."

Medium Performance Message Example: "Hello [Name]! Mid-week check results (as of Thursday morning): You've reached more than half of this week's goal (25,000 steps). If you walk around the neighborhood on weekends, you'll be able to reach your goal quickly. [Name]! I'm cheering for you to secure 500 points. I'll visit again next week. Thank you."

High Performance Message Example: "Hello [Name]! You've already walked more than 25,000 steps even though it hasn't been a week yet. Excellent work. You've secured 500 points. Congratulations. Take wonderful photos while walking around the neighborhood on weekends~ I'll visit again next week. Thank you.
