# Supplementary material for: Personalized Mobile App–Based Messaging and Step Tracking for Improving Walking Behavior in Adults: Randomized Controlled Trial
Source: JMIR Mhealth Uhealth. 2026 Sep 17;14:e84430. doi: 10.2196/84430 (PMC13631492; doi:10.2196/84430)
Supplement: Supplementary file 3 [file mhealth_v14i1e84430_app3.docx]

Multimedia Appendix 3. Participant/nonparticipant comparison and attrition bias assessment.

**Table S1. Baseline comparison between intervention and control groups (actual participants).**

| Variable | Intervention group (N=67), n (%) or mean (SD) | Control group (N=74), n (%) or mean (SD) | χ² or t | *P* value |
| --- | --- | --- | --- | --- |
| **Gender** |  |  |  |  |
| Men | 19 (28) | 29 (39) | 1.837 | .18 |
| Women | 48 (72) | 45 (60) |  |  |
| **Age group** |  |  |  |  |
| 20s | 9 (13) | 5 (7) | 9.118 | .06 |
| 30s | 8 (12) | 14 (19) |  |  |
| 40s | 16 (24) | 31 (42) |  |  |
| 50s | 27 (40) | 18 (24) |  |  |

^a^ Baseline step count (week 1) comparison: Intervention 60,940 (31,708); Control 57,217 (26,610); t=0.748, P=.46.

**Table S2. Attrition bias assessment: participants vs non-participants.**

| Variable | Participants (N=141), n (%) | Non-participants (N=33), n (%) | χ² | *P* value |
| --- | --- | --- | --- | --- |
| **Gender** |  |  |  |  |
| Men | 48 (34) | 12 (36) | 0.064 | .80 |
| Women | 93 (66) | 21 (64) |  |  |
| **Age group** |  |  |  |  |
| 20s | 14 (9.9) | 3 (9) | 2.389 | .67 |
| 30s | 22 (15.6) | 4 (12) |  |  |
| 40s | 47 (33.3) | 11 (33) |  |  |
| 50s | 45 (31.9) | 9 (27) |  |  |
