# Supplementary material for: Personalized Mobile App–Based Messaging and Step Tracking for Improving Walking Behavior in Adults: Randomized Controlled Trial
Source: JMIR Mhealth Uhealth. 2026 Sep 17;14:e84430. doi: 10.2196/84430 (PMC13631492; doi:10.2196/84430)
Supplement: Supplementary file 4 [file mhealth_v14i1e84430_app4.docx]

Multimedia Appendix 4. Detailed weekly step count data and weekly mean step counts.

**Table S3. Weekly goal achievement rates (25,000 Steps).**

| Week | Intervention group (N=67), n (%) | Control group (N=74), n (%) | Difference percentage points |
| --- | --- | --- | --- |
| 1 | 62/67 (93) | 69/74 (93) | 0 |
| 2 | 62/67 (93) | 67/74 (91) | 2 |
| 3 | 58/67 (87) | 64/74 (87) | 0 |
| 4 | 61/67 (91) | 66/74 (89) | 2 |
| 5 | 61/67 (91) | 65/74 (88) | 3 |
| 6 | 61/67 (91) | 63/74 (85) | 6 |
| 7 | 58/67 (87) | 62/74 (84) | 3 |
| 8 | 58/67 (87) | 63/74 (85) | 2 |
| 9 | 56/67 (84) | 67/74 (91) | -7 |
| 10 | 56/67 (84) | 66/74 (89) | -5 |
| 11 | 54/67 (81) | 61/74 (82) | -1 |
| 12 | 54/67 (81) | 56/74 (76) | 5 |
| Overall | 58/67 (87) | 64/74 (87) | 0 |
| ^a^ N = 141 participants | | | |

**Table S4. Weekly mean step count (mean [SD]).**

| Week | | Intervention group, mean (SD) |  | Control group, mean (SD) | Difference (intervention - control), steps |
| --- | --- | --- | --- | --- | --- |
| 1 | | 57,217 (26,610) |  | 60,940 (31,708) | -3,723 |
| 2 | | 56,605 (26,682) |  | 62,450 (32,697) | -5,845 |
| 3 | | 51,464 (24,187) |  | 54,107 (29,203) | -2,642 |
| 4 | | 60,622 (27,334) |  | 58,369 (30,507) | 2,253 |
| 5 | | 56,369 (24,104) |  | 57,480 (29,994) | -1,112 |
| 6 | | 54,064 (20,687) |  | 54,981 (31,607) | -917 |
| 7 | | 55,468 (22,389) |  | 56,318 (31,168) | -851 |
| 8 | | 54,685 (22,843) |  | 59,664 (33,701) | -4,978 |
| 9 | | 58,751 (26,566) |  | 64,247 (33,599) | -5,496 |
| 10 | | 52,417 (25,164) |  | 59,742 (30,526) | -7,324 |
| 11 | | 54,209 (24,273) |  | 60,669 (32,643) | -6,461 |
| 12 | | 49,067 (23,242) |  | 54,808 (29,684) | -5,741 |
| Overall | | 55,082 |  | 58,660 | -3,579 |
|  | ^a^ N = 141 participants | | | | |
