# Supplementary material for: Personalized Mobile App–Based Messaging and Step Tracking for Improving Walking Behavior in Adults: Randomized Controlled Trial
Source: JMIR Mhealth Uhealth. 2026 Sep 17;14:e84430. doi: 10.2196/84430 (PMC13631492; doi:10.2196/84430)
Supplement: Supplementary file 5 [file mhealth_v14i1e84430_app5.docx]

Multimedia Appendix 5. Additional survival analysis details

**Table S5 Consecutive 2-week goal achievement failure events**

| Outcome | Intervention group(N=67) | Control group(N=74) | Total(N=141) |
| --- | --- | --- | --- |
| Consecutive 2-week goal achievement failure events, n (%) | 23 (34%) | 27 (37%) | 50 (35.5%) |
| Mean failure event, weeks | 4.9 | 4.7 | 4.8 |
| 12-week cumulative survival rate, % | 65.7 | 63.5 | 64.5 |

**Table S6 Kaplan-Meier survival analysis results**

| Group | Mean survival time, weeks (SE) | 95% CI | Median | Log‑rank statistic (*χ²*) | *P value* |
| --- | --- | --- | --- | --- | --- |
| Control group | 11.092(0.082) | 10.93–11.25 | Not Reached | 9.938 | .002 |
| Intervention group | 11.425(0.069) | 11.29–11.56 | Not Reached |  |  |
| Total | 11.259(0.053) | 11.15–11.36 | Not Reached |  |  |
| ^b^Note. The median time to the event was not reached within the 12‑week follow‑up period. | | | | | |
